# Investigation of Equine In Vivo and In Vitro Derived Metabolites of the Selective Androgen Receptor Modulator (SARM) ACP-105 for Improved Doping Control

**DOI:** 10.3390/metabo11020085

**Published:** 2021-02-01

**Authors:** Malin Nilsson Broberg, Heather Knych, Ulf Bondesson, Curt Pettersson, Scott Stanley, Mario Thevis, Mikael Hedeland

**Affiliations:** 1 Department of Medicinal Chemistry, Uppsala University, Box 574, SE-75123 Uppsala, Sweden; malin.broberg@ilk.uu.se (M.N.B.); ulf.bondesson@ilk.uu.se (U.B.); curt.pettersson@ilk.uu.se (C.P.); 2 Kenneth L. Maddy Equine Analytical Pharmacology Laboratory, School of Veterinary Medicine, University of California, Davis, CA 95616, USA; hkknych@ucdavis.edu; 3 Department of Chemistry, Environment and Feed Hygiene, National Veterinary Institute (SVA), SE-75189 Uppsala, Sweden; 4 Gluck Equine Research Center, University of Kentucky, Lexington, KY 40546, USA; sdst245@uky.edu; 5 Institute of Biochemistry, Center for Preventive Doping Research, German Sport University, 50933 Cologne, Germany; thevis@dshs-koeln.de

**Keywords:** SARM, Selective Androgen Receptor Modulator, ACP-105, mass spectrometry, doping, horse, metabolites, microsomes, *Cunninghamella elegans*

## Abstract

Selective Androgen Receptor Modulators (SARMs) have anabolic properties but less adverse effects than anabolic androgenic steroids. They are prohibited in both equine and human sports and there have been several cases of SARMs findings reported over the last few years. The aim of this study was to investigate the metabolite profile of the SARM ACP-105 (2-chloro-4-[(3-endo)-3-hydroxy-3-methyl-8-azabicyclo[3.2.1]oct-8-yl]-3-methylbenzonitrile) in order to find analytical targets for doping control. Oral administration of ACP-105 was performed in horses, where blood and urine samples were collected over a time period of 96 h. The in vivo samples were compared with five in vitro incubation models encompassing *Cunninghamella elegans*, microsomes and S9 fractions of both human and equine origin. The analyses were performed using ultra-high performance liquid chromatography coupled to high resolution Q Exactive^TM^ Orbitrap^TM^ mass spectrometry (UHPLC-HRMS). A total of 21 metabolites were tentatively identified from the in vivo experiments, of which several novel glucuronides were detected in plasma and urine. In hydrolyzed urine, hydroxylated metabolites dominated. The in vitro models yielded several biotransformation products, including a number of monohydroxylated metabolites matching the in vivo results. The suggested analytical target for equine doping control in plasma is a dihydroxylated metabolite with a net loss of two hydrogens. In urine, the suggested targets are two monohydroxylated metabolites after hydrolysis with β-glucuronidase, selected both due to prolongation of the detection time and the availability of reference material from the in vitro models.

## 1. Introduction

Selective Androgen Receptor Modulators (SARMs) are a novel class of compounds that are androgen receptor ligands with anabolic properties similar to those of anabolic steroids in muscle and bone, but with a milder profile of adverse effects, since they are more tissue selective [1,2,3]. There have been several clinical trials involving SARMs for treatment of different diseases such as cachexia and sarcopenia, but so far none of them have been approved in a pharmaceutical product [3,4,5]. The pharmacological effects and limited side effects make them tempting to use as doping agents and they have been on the World Anti-Doping Agency’s Prohibited List since 2008 [6,7]. SARMs are also included in the regulations of the International Federation of Horseracing Authorities (IFHA) and are prohibited both in and out of equine competition [8]. Many SARMs are readily available to purchase online and there have been several doping cases involving this substance class in both humans and horses in the past few years [9,10,11,12,13].

Knowledge of the metabolite profile is important to prolong the detection window of doping substances, since metabolites may be present in biological samples for a longer time than the parent compound [14,15]. Besides in vivo administration studies, in vitro methods, such as microsomal and S9 fraction incubations, are of interest from the perspective of prediction of metabolite patterns. Furthermore, in vitro generated metabolites from microsome incubations may be used as reference material in both equine and human doping control in accordance with the International Laboratory Accreditation Cooperation document ILAC-G7 Accreditation Requirements and Operating Criteria for Horseracing Laboratories and the World Anti-Doping Agency’s International Standard for Laboratories [16,17]. In addition, the fungus *Cunninghamella elegans* has been shown to produce phase I metabolites similar to those of both horses and humans, but the metabolite profile is often more extensive [18,19,20]. It has also been possible to scale up the method to purify metabolites for use as reference substances [21].

The SARM ACP-105 (2-chloro-4-[(3-endo)-3-hydroxy-3-methyl-8-azabicyclo[3.2.1]oct-8-yl]-3-methylbenzonitrile) was first described in 2008 and structurally characterized with mass spectrometry in 2013 [22,23]. An in vivo study performed in rats showed that the detected metabolites in urine consisted of several structural isomers of monohydroxylated and dihydroxylated ACP-105 after β-glucuronidase hydrolysis [14]. A recent equine in vivo study of metabolites from ACP-105 in urine has also been published, where several phase I metabolites were presented, such as hydroxylated and dehydrated products, but no phase II metabolites such as glucuronides were described [24]. However, there is no published information on neither the phase II metabolism nor the plasma metabolite pattern of this compound in the horse and suitable analytical targets in these matrices are yet to be determined.

The aim of this study was to investigate the in vivo metabolite profile in equine plasma and urine after oral administration of ACP-105 and compare with five relevant in vitro models for possible identification and production of analytical reference material. The combined information was used to identify suitable analytical targets that can be used in doping control with ultra-high performance liquid chromatography–high resolution mass spectrometry (UHPLC-HRMS).

## 2. Results and Discussion

The identification and time profile analysis of metabolites formed from ACP-105 in both the in vivo and in vitro experiments were performed by UHPLC-HRMS full scan and MS/MS product ion scan modes. The reference standard RAD-140 was analyzed in the beginning and at the end of each batch. The acceptance criteria for batch approval were <0.02 min drift in retention time and *m*/*z* error < 5 ppm. Due to the qualitative properties of this study, data regarding replicates are not presented, but each biological sample has been analyzed a minimum of five times with consistent results.

### 2.1. Detection of Parent ACP-105

**Figure 1:**
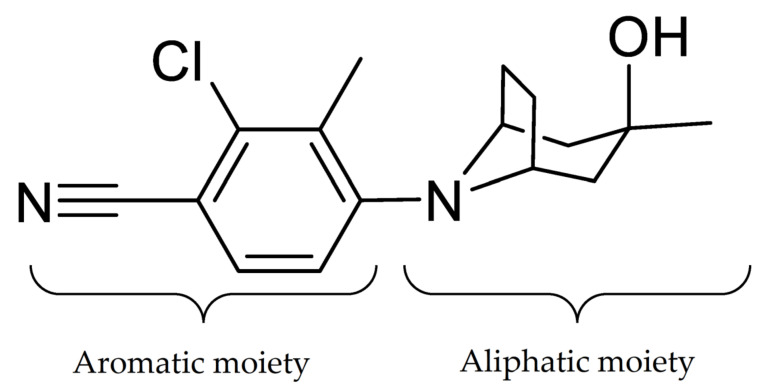
The structure of ACP-105 (2-chloro-4-[(3-endo)-3-hydroxy-3-methyl-8-azabicyclo[3.2.1]oct-8-yl]-3-methylbenzonitrile).

**Figure 2:**
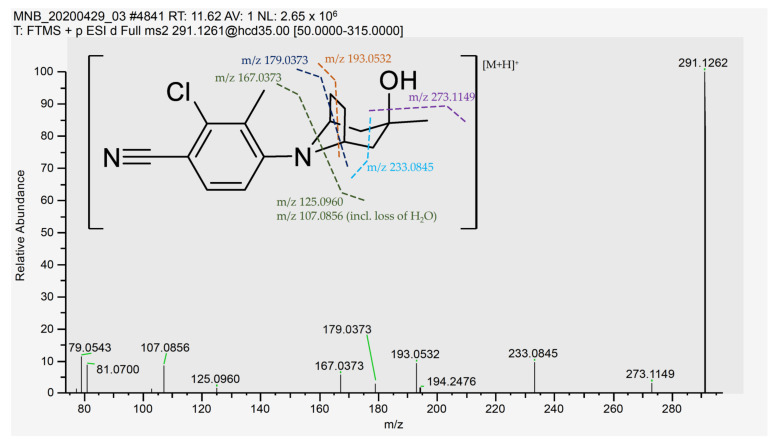
MS/MS spectrum and suggested cleavage sites for the product ions of ACP-105 with the pre-cursor ion [C_16_H_20_ClN_2_O]^+^ with *m*/*z* 291.1262. The suggested cleavage sites do not represent the final hydrogen distribution between the detected fragment and the neutral loss for all fragments, full information available in Table A1 in Appendix A.

Initially, a standard solution of ACP-105 was analyzed and the retention time, ionization and MS/MS fragmentation were studied. ACP-105 was observed at a retention time of 11.59 min and the main ion in positive mode was C_16_H_20_ClN_2_O^+^ at *m*/*z* 291.1262 corresponding to [M + H]^+^. The structure of ACP-105 is displayed in Figure 1. The aromatic moiety consists of 2-chloro-3-methylbenzonitrile and the aliphatic moiety consists of 3-methyl-8-azabicyclo[3.2.1]octan-3-ol. As seen in Figure 2 and Table A1 in Appendix A, in MS/MS mode, the main product ions were *m*/*z* 273.1149 representing the loss of H_2_O, *m*/*z* 233.0845 with the neutral loss of C_3_H_6_O that occurs due to a cleavage of the aliphatic moiety along with both the *m*/*z* 193.0532 and *m*/*z* 179.0373 fragments. The species *m*/*z* 167.0373 corresponds to a fragment with the aromatic moiety intact. The *m*/*z* 125.0960 with the neutral loss of C_8_H_7_ClN_2_ corresponds to a fragment formed by a loss of mainly the aromatic moiety and *m*/*z* 107.0856 represents the same fragment with an additional loss of H_2_O. The structural elucidation of ACP-105 is consistent with studies previously performed by Thevis et al. where they also made tentative identifications of a variety of in vivo derived hydroxylated metabolites [14,23].

ACP-105 was detected in plasma for up to 24 h after administration. The longest detection time in untreated urine was 24 h, but after hydrolysis with β-glucuronidase the detection time was increased to 48 h.

### 2.2. In Vivo Metabolites

**Figure 3:**
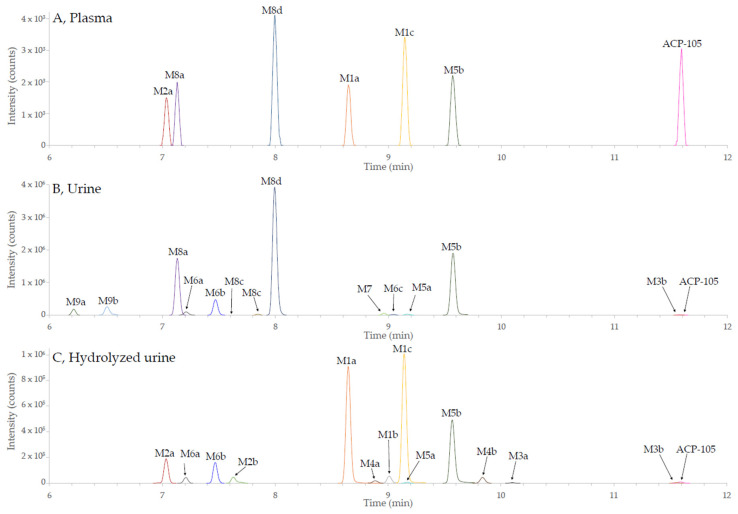
Combined extracted ion chromatograms of the metabolites of ACP-105 fulfilling the set criteria from the UHPLC-HRMS analysis. A full description of the metabolites and their denotations can be found in Table A1 in Appendix A. (**A**) metabolites in plasma 12 h after administration; (**B**) metabolites in urine 24 h after administration and; (**C**) metabolites in hydrolyzed urine 24 h after administration. The metabolite labels represent the following metabolic transformations: monohydroxylation (M1), dihydroxylation (M2), loss of 2H (M3), loss of 2H and monohydroxylation (M4), loss of 2H and dihydroxylation (M5), loss of 2H and trihydroxylation (M6), glucuronidation (M7), monohydroxylation and glucuronidation (M8) and dihydroxylation and glucuronidation (M9).

ACP-105 is excreted to a small extent in unchanged form, making the analysis of metabolites of high interest for doping control. In total, 21 phase I and phase II metabolites could be tentatively identified in plasma, urine and hydrolyzed urine as demonstrated in Figure 3. The acceptance criteria for the tentative identification were: mass error < 5 ppm from the calculated theoretical value, retention time drift < 0.02 min between the different sample matrices for the same metabolite, chromatographic peaks consisting of a minimum of five scans and MS/MS spectrum with product ions matching the suggested structure of the metabolite. There were also signs of several additional structural isomers of some metabolites but the data did not meet the mentioned criteria and they are thereby not presented. In Figure 3, only the extracted ion chromatograms (isolation width *m*/*z* < 2 ppm) are reported for the metabolites of ACP-105 that have been tentatively identified by the set criteria described above. Due to this fact, other substances present in the matrices, such as endogenous compounds, are not displayed.

All tentatively identified metabolites and detailed information about their detectability in plasma, urine and hydrolyzed urine together with information about the fragmentation can be found in Table A1 in Appendix A. Mass spectra and suggested fragments for all tentatively identified metabolites can be found in Supplementary Materials Figure S1.

The phase I metabolic transformations resulted in mono-, di- and trihydroxylated forms also in some cases in combination with a net loss of two hydrogens. The loss of two hydrogens could be observed in the aliphatic structure, indicating a double bond formation. The mechanism could not be elucidated, but possible reactions include loss of water after hydroxylation, oxidation of an alcohol or dehydrogenation. The net loss of two hydrogens was also observed in the previous in vivo study and explained as a result of dehydration [24].

Three monohydroxylated metabolites, M1a-c (C_16_H_19_ClN_2_O_2_) were tentatively identified and detected in hydrolyzed urine where they had the longest detection time. Metabolites M1a and M1c were also detected in plasma, but not in untreated urine, indicating that glucuronic acid conjugation had taken place. 

Two dihydroxylated metabolites, M2a-b (C_16_H_19_ClN_2_O_3_) were detected in hydrolyzed urine, however, metabolite M2a could also be observed in untreated urine and plasma. Two metabolites formed by a net loss of two hydrogens, M3a-b (C_16_H_17_ClN_2_O) could be tentatively identified in hydrolyzed urine. Metabolite M3b could also be seen in untreated urine, but the detection time was longer in hydrolyzed urine. 

Several phase I metabolites formed by a combination of different metabolic transformations were also found, such as two monohydroxylated metabolites with a net loss of two hydrogens, M4a-b (C_16_H_17_ClN_2_O_2_), that could mainly be found in hydrolyzed urine. Two dihydroxylated metabolites with a net loss of two hydrogens, M5a-b (C_16_H_17_ClN_2_O_3_) were also tentatively identified. M5a could be found both in untreated as well as in hydrolyzed urine. Metabolite M5b could be found in all analyzed matrices, but with a shorter detection time in plasma. An additional three trihydroxylated metabolites with a net loss of two hydrogens, M6a-c (C_16_H_17_ClN_2_O_4_) were also detected in both untreated and hydrolyzed urine.

Furthermore, seven glucuronides were detected and summarized in Table A1 in Appendix A, out of which, one was the directly glucuronidated parent compound, metabolite M7 (C_22_H_27_ClN_2_O_7_) that was mainly detected in untreated urine. Four glucuronidated forms of monohydroxylated metabolites, M8a-M8d (C_22_H_27_ClN_2_O_8_) were also observed. Metabolites M8a and M8d could be seen in all matrices but had the longest detection time in untreated urine. Metabolites M8b and M8c could only be detected in untreated urine. Two glucuronidated forms of dihydroxylated metabolites, M9a-M9b (C_22_H_27_ClN_2_O_8_) were detected mainly in untreated urine. All these metabolites had a higher intensity and longer detection time in untreated urine than in hydrolyzed urine, supporting the tentative identification of these metabolites.

The retention order of the metabolites and the parent compound matched the expected polarity differences based on the tentative identifications (Figure 3 and Table A1 in Appendix A), e.g., monohydroxylated metabolites (M1a-c) have a longer retention time than dihydroxylated metabolites (M2a-b).

#### Structural Elucidation of Major Metabolites

**Figure 4:**
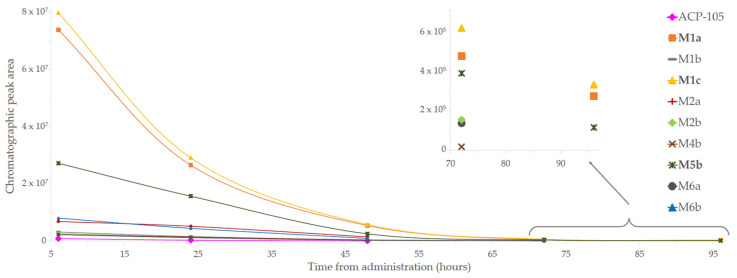
Time profile of the metabolites from ACP-105 present in hydrolyzed urine. The metabolites M1a, M1c and M5b were detected up to 96 h. The figure shows the time from administration of ACP-105 (*x*-axis) and the chromatographic peak area (*y*-axis).

The major metabolites were selected based on their detectability in the different matrices. In plasma, the monohydroxylated metabolite M1c together with the monohydroxylated glucuronides M8a and M8d had the highest intensities in the investigated time interval, see Figure 3 and Table A1. In untreated urine, the dihydroxylated metabolite formed by a net loss of two hydrogens, M5b, together with metabolite M8a and M8d were detected with the highest intensities over time. The time profiles for the metabolites present in hydrolyzed urine are shown in Figure 4. The metabolites with the highest intensities and with a detectability of at least 96 h after administration in hydrolyzed urine were the monohydroxylated metabolites M1a and M1c together with M5b. This combined information was used to select the five metabolites M1a, M1c, M5b, M8a and M8d for further structural characterization.

**Figure 5:**
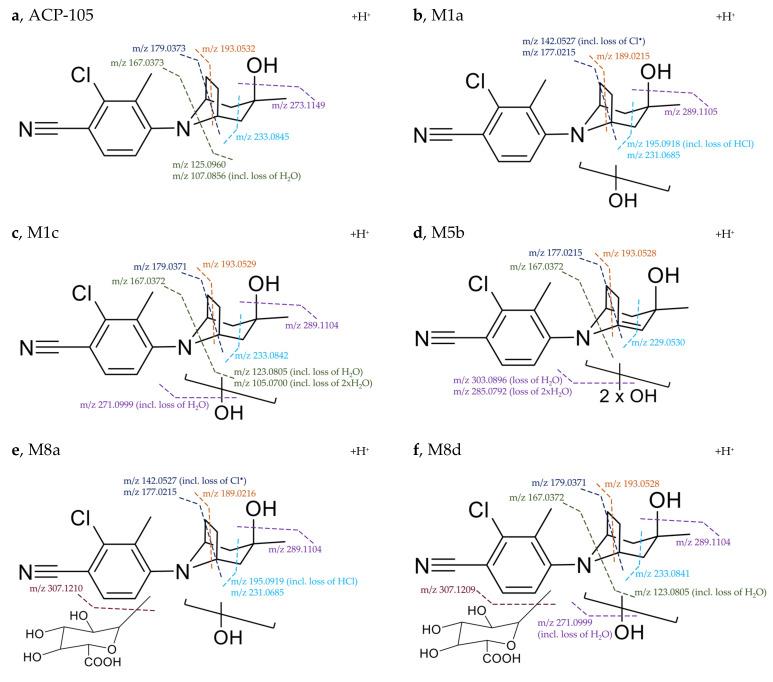
Suggested fragmentation pattern of the major metabolites in comparison with ACP-105. (**a**) Parent ACP-105—precursor ion [C_16_H_20_ClN_2_O]^+^ at *m*/*z* 291.1262; (**b**) monohydroxylated metabolite M1a—precursor ion [C_16_H_20_ClN_2_O_2_]^+^ at *m*/*z* 307.1209; (**c**) monohydroxylated metabolite M1c—precursor ion [C_16_H_20_ClN_2_O_2_]^+^ at *m*/*z* 307.1209; (**d**) dihydroxylated metabolite with formed double bond M5b—precursor ion [C_16_H_18_ClN_2_O_3_]^+^ at *m*/*z* 321.1003. The exact position of the formed double bond within the aliphatic ring structure is not known, but it will change the previous chair formation; (**e**) monohydroxylated and glucuronidated metabolite M8a—precursor ion [C_22_H_28_ClN_2_O_8_]^+^ at *m*/*z* 483.1532; (**f**) monohydroxylated and glucuronidated metabolite M8d—precursor ion [C_22_H_28_ClN_2_O_8_]^+^ at *m*/*z* 483.1530.

The diagnostic fragments used for the structural elucidation of the five most abundant metabolites are shown in Figure 5. For the monohydroxylated metabolite M1a bearing one additional oxygen in comparison to ACP-105, the fragmentation indicated that the hydroxylation had taken place on the aliphatic moiety of the structure. The presence of *m*/*z* 142.0527, which represents the aromatic moiety with a loss of Cl^•^ indicates that no metabolic transformations have occurred on this part of the molecule. The product ions at *m*/*z* 231.0685 and *m*/*z* 177.0215 contained two hydrogens less than the corresponding ACP-105 fragments, suggesting a loss of H_2_O somewhere in the ring structure of the aliphatic moiety. This indicates that the hydroxylation had occurred somewhere on the aliphatic ring and not on the methyl group.

The monohydroxylated metabolite M1c produced the product ions *m*/*z* 233.0842, *m*/*z* 193.0529, *m*/*z* 179.0371 and *m*/*z* 167.0372 that matched the ACP-105 fragments and indicated that the aromatic moiety is metabolically unchanged, suggesting that the hydroxylation had taken place on the aliphatic moiety in this case as well. Furthermore, the unchanged aliphatic ring fragments *m*/*z* 179.0371 and *m*/*z* 167.0372 indicated that the hydroxylation had most likely taken place on the methyl group of the aliphatic moiety as suggested in previous studies [14].

The dihydroxylated metabolite with a net loss of two hydrogens, M5b also produced the fragment *m*/*z* 167.0372, indicating that the aromatic moiety was metabolically unchanged and that the metabolic transformations had taken place on the aliphatic moiety. Fragments *m*/*z* 177.0215 and *m*/*z* 229.0530 were two and four units lower, in comparison to the corresponding product ions of ACP-105. This suggests that at least two metabolic transformations had taken place in the ring structure of the aliphatic moiety, probably due to the loss of H_2_O and formation of a double bond.

All diagnostic fragments from the monohydroxylated metabolite M1a matched those of the monohydroxylated glucuronide M8a. Together with the increased intensity of M1a and decreased intensity of M8a after hydrolysis, M8a is most likely a phase II metabolite of M1a. The same relation can be observed for the monohydroxylated metabolite M1c and the monohydroxylated glucuronide metabolite M8d. This clearly demonstrates the value of β-glucuronidase hydrolysis in the sample preparation of urine for increased intensity and extended detection times of illicit use of ACP-105.

### 2.3. Metabolites from C. elegans Incubations

**Figure 6:**
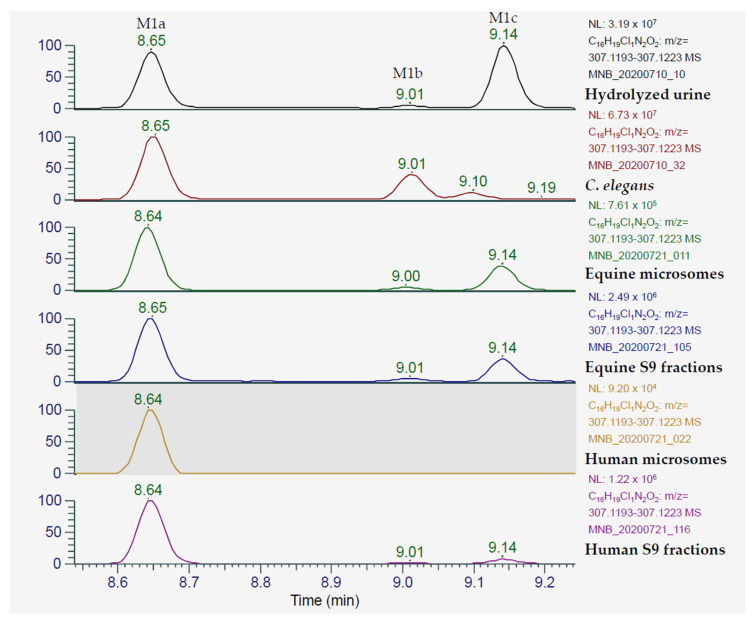
Extracted ion chromatograms of monohydroxylated ACP-105 [C_16_H_20_ClN_2_O_2_]^+^ that present the metabolites detected in hydrolyzed urine, *C. elegans* incubates and incubates of equine and human microsomes and S9 fractions. In hydrolyzed urine, the peak at 8.65 min represents metabolite M1a, the peak at 9.01 min represents the metabolite M1b and the peak at 9.14 min represents the metabolite M1c. The figure shows the retention time in minutes (*x*-axis) and the relative mass spectrometric intensity (*y*-axis).

The metabolite profile of ACP-105 in *C. elegans* incubates encompasses a range of different phase I and phase II products of interest, such as monohydroxylated, dihydroxylated and glycosylated forms of ACP-105. The absence of glucuronides was expected, since it has previously been shown that this fungus produces glycosides instead [21]. The most relevant findings were that it produced several structural isomers of monohydroxylated ACP-105 including two with retention time and product ion spectrum matching those of M1a and M1b from the in vivo study including the set criteria for tentative identification, see Figure 6. M1a is a major metabolite in equine plasma with a detection time of 24 h. M1a is also tentatively identified in hydrolyzed urine with a detection time exceeding 96 h and is an interesting target for doping analysis, making *C. elegans* incubations a promising method to produce analytical reference material.

### 2.4. Metabolites from Microsome and S9 Fraction Incubations

Both the equine microsomes and the equine S9 fraction produced the monohydroxylated metabolites M1a, M1b and M1c in a similar ratio as in hydrolyzed urine, see Figure 6. Several additional metabolites were detected, such as the dihydroxylated metabolite with a net loss of two hydrogens, M5b that could be detected in the equine S9 fraction, but at a low intensity. Moreover, the glucuronide M7 and the monohydroxylated glucuronide M8d could be tentatively identified in equine and human microsome and S9 fraction incubates, but at a low intensity and the data is therefore not presented. The structural elucidation of the metabolites M1a-M1c was performed by comparing the MS/MS spectra with spectra from the in vivo analysis along with matching retention times. The results met the set criteria for tentative identification for the abovementioned metabolites of ACP-105. Thus, this study has shown the potential to use equine microsome incubates as reference material for ACP-105 doping analysis.

In addition to the equine microsome and S9 fraction analysis, an inter-species comparison with human microsomes and S9 fractions was performed. This is of interest not only to compare the metabolite profiles, but also for the future possibility to compare the results with those of actual human doping control samples. The human microsome and S9 fraction incubates both produced the monohydroxylated M1a. In the S9 fraction incubates M1c was also tentatively identified, this was done by comparing MS/MS spectra and retention time with those of the in vivo analysis for both metabolites. The S9 fractions also potentially produced metabolite M1b, but it could not be confirmed in this analysis due to low intensity of the MS signal.

### 2.5. Suggested Analytical Target for ACP-105 Equine Doping Analysis

The comparison of the plasma, urine and hydrolyzed urine samples with the incubation systems shows the importance of both the in vivo and in vitro models. To confirm the presence of illicit substances in the doping analysis results, reference material is needed—this is commonly created by organic synthesis, purified and characterized by NMR. Synthesis methods can be complex and take time to develop. The methods shown in this paper provide several additional solutions. Samples from microsome incubations with the substance can be used as reference material in accordance with the current guidelines for equine and human doping analysis [16,17]. *C. elegans* incubations can be scaled up and purified and characterized reference substance can be produced on a larger scale.

**Figure 7:**
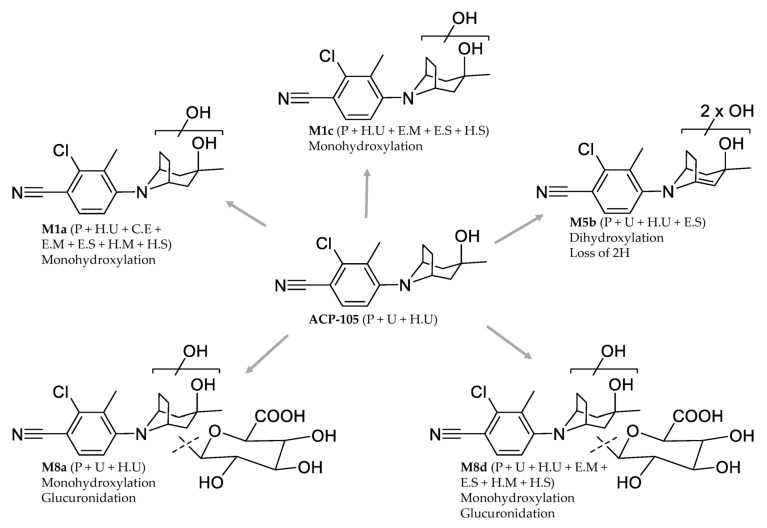
The proposed structures and location of metabolic transformations of the major metabolites from ACP-105. The information within the brackets indicates if they are present in plasma (P) urine (U), hydrolyzed urine (H.U) and incubates with *C. elegans* (C.E), equine microsomes (E.M), equine S9 fractions (E.S), human microsomes (H.M) and human S9 fractions (H.S). For metabolite M5b, the exact position of the double bond within the aliphatic ring structure is not known.

In this study, five major metabolites were tentatively identified as observed in Figure 7. The monohydroxylated metabolites M1a and M1c together with the dihydroxylated metabolite with a loss of two hydrogens, M5b are clearly detectable at 96 h in hydrolyzed urine, which can be seen in Figure 4, showing that the detection time is even longer than was tested in this study. The use of the monohydroxylated metabolites M1a and M1c is recommended as analytical targets in doping analysis after hydrolysis of urine. This is due to the great increase in detection time after ACP-105 administration in combination with the availability of reference material from several of the in vitro models. In plasma, metabolite M5b is recommended as an analytical target, but the glucuronidated monohydroxlated metabolites M8a and M8d could also be used since they have a similar detection level.

## 3. Materials and Methods

### 3.1. Chemicals

ACP-105 (2-chloro-4-[(3-endo)-3-hydroxy-3-methyl-8-azabicyclo[3.2.1]oct-8-yl]-3-methylbenzo-nitrile) (98.37%) for the administration part of the study was purchased from ChemScene (Monmouth Junction, NJ, USA). RAD-140 (2-chloro-4-[[(1R,2S)-1-[5-(4-cyanophenyl)-1,3,4-oxadiazol-2-yl]-2-hydroxypropyl]amino]-3-methylbenzonitrile) and ACP-105 for analysis were purchased from MedChemExpress (Monmouth Junction, NJ, USA) and LGD-4033 (4-[(2R)-2-[(1R)-2,2,2-trifluoro-1-hydroxyethyl]-1-pyrrolidinyl]-2-(trifluoromethyl)-benzonitrile) was purchased from Toronto Research Chemicals (North York, ON, Canada). β-nicotinamide adenine dinucleotide phosphate sodium salt hydrate (β-NADPH), uridine 5′-diphosphoglucuronic acid trisodium salt (UDPGA) and sabouraud dextrose broth were purchased from Sigma-Aldrich (St. Louis, MO, USA). Human pooled liver microsomes, human S9 fractions, individual equine microsomes and equine S9 fractions all at the concentration of 20 mg_·_ml^−1^ were purchased from Sekisui Xenotech (Kansas City, KS, USA). β-glucuronidase from E. coli K12 (80 U_·_mg^−1^ at 25 °C) was purchased from Roche (Basel, Switzerland). Formic acid (Optima LC-MS grade) and Pierce^TM^ electrospray ionization (ESI) Positive/Negative Ion Calibration Solution were purchased from Thermo Fisher Scientific (Waltham, MA, USA). *Cunninghamella elegans* ATCC 9245 was purchased from LGC Promochem (Borås, Sweden). Sodium chloride solution (0.86–0.90%) and agar plates (mycological peptone (10 g_·_L^−1^), dextrose (40 g_·_L^−1^) and agar (15 g_·_L^−1^)) were produced in house at the National Veterinary Institute (SVA, Uppsala, Sweden). Purified water was obtained using Milli-Q^®^ Advantage A10 with a 0.22-µm filter from Millipore (Burlington, MA, USA). All other chemicals and solvents were of analytical grade or higher.

### 3.2. Drug Administration and Sample Collection

Out of six University of California-owned adult Thoroughbred horses (3 mares and 3 geldings; age: 4–8 years; weight: 515.5–569.5 kg), one mare (horse A) and one gelding (horse B) were randomly selected using a computerized random number generator for drug administration. Before beginning the study, horses were determined healthy and free of disease by physical examination, complete blood count and a serum biochemistry panel that included aspartate aminotransferase, creatinine phosphokinase, alkaline phosphatase, total bilirubin, sorbitol dehydrogenase, blood urea nitrogen and creatinine. Blood analyses were performed by the Clinical Diagnostic Laboratories at the William R. Pritchard Veterinary Medical Teaching Hospital of the University of California, Davis, using their standard protocols. The horses did not receive any medications for at least two weeks prior to commencement of this study. Food and water were available ad libitum throughout the duration of the study. This study was approved by the Institutional Animal Care and Use Committee of the University of California, Davis (Protocol #20319, date of approval 11 January 2020).

ACP-105 was weighed and suspended in 0.9% NaCl and DMSO (25% of total volume) and administered orally at a dose of 0.05 mg_·_kg^−1^ ACP-105 into the back of the oral cavity using an oral dosing syringe. A A14-gauge catheter was aseptically placed in one external jugular vein for sample collection. Blood samples were collected at time 0 (immediately prior to ACP-105 administration) and at 15, 30, and 45 min, and 1, 1.5, 2, 2.5, 3, 4, 5, 6, 8, 12, 24, 36, 48, 72 and 96 h post ACP-105 administration. Prior to drawing each blood sample, 10 mL of blood was aspirated and discarded from the catheter and T-Port extension set (combined internal volume < 2 mL). The catheter was flushed with 10 mL of a dilute heparinized saline solution (10 IU_·_mL^−1^) following each sampling time. The jugular vein catheter, used for sample collection, was removed following the 24-h sample collection and the blood samples at 36, 48, 72 and 96 h post ACP-105 administration were collected via direct venipuncture. Blood samples were collected into ethylenediaminetetraacetic acid (EDTA) containing blood tubes and placed on ice prior to centrifugation at 1620× *g* for 10 min at 4 °C. Plasma was then immediately transferred into storage cryovials (Phenix Research Products, Chandler, NC, USA) and stored at −20 °C until analysis. Urine samples were collected between 6 and 7, 24, 48, 72 and 96 h post ACP-105 administration. Urine samples were collected by free catch and were stored at −20 °C until analysis.

### 3.3. Urine Sample Preparation

#### 3.3.1. Sample Dilution

Urine samples were centrifuged in Eppendorf tubes at 11,500× *g* for 10 min in a Sepatech Biofuge 15 (Heraeus, Hanau, Germany) and the supernatant was diluted 1:1 with aqueous formic acid (0.1%) and transferred to a vial for analysis. 

#### 3.3.2. Solid-Phase Extraction with HLB 

The urine samples were prepared with a generic method by first diluting 2.0 mL urine with 2.0 mL aqueous formic acid (0.1%). Oasis HLB 60 mg solid phase extraction (SPE) cartridges from Waters (Milford, MA, USA) were conditioned with 2.0 mL MeOH and 2.0 mL aqueous formic acid (0.1%). The samples were loaded on to the cartridge and washed with 2.0 mL 5% MeOH in water and thereafter eluted using 3.0 mL MeOH. The solvent was gently evaporated at 50 °C under a stream of nitrogen. The samples were reconstituted in 600 µL aqueous formic acid (0.1%), centrifuged 11,500× *g* for 10 min and transferred to vials for analysis.

#### 3.3.3. Hydrolysis with β-Glucuronidase

Urine samples (2.0 mL) were added to 2.0 mL phosphate buffer (0.1 M, pH 6.10) and 100 µL β-glucuronidase was added. The samples were placed in a heating bath and incubated at 50 °C for 2 h. They were cooled to room temperature and thereafter extracted using the SPE method described in Section 3.3.2. The samples were reconstituted in 700 µL aqueous formic acid (0.1%) and transferred to vials for analysis.

### 3.4. Plasma Sample Preparation

Plasma precipitation was performed by transferring 200 µL of plasma samples to an Eppendorf tube. An amount of 800 µL ice cold acetonitrile was added and the Eppendorf tubes were mixed using a vortex mixer and stored at 5 °C for 20 min. The samples were centrifuged at 11,500× *g* for 10 min and 800 µL of the supernatant was transferred to a new Eppendorf tube and evaporated to dryness using a vacuum centrifuge Christ RVC 2-8 (Martin Christ, Osterode am Harz, Germany). The samples were thereafter reconstituted in 200 µL aqueous formic acid (0.1%) and transferred to vials for analysis.

### 3.5. Microsomes and S9 Fraction Incubation

Potassium phosphate buffer (PB) pH 7.4, 50 mM was prepared from 1.0 M KH_2_PO_4_, and 1.0 M K_2_HPO_4_ with the addition of 5 mM MgCl_2_. To a 1.5-mL Eppendorf tube, the following was added; 25 µL PB, 10 µL ACP-105 solution (10 µM, 2.5% MeOH in PB), 10 µL β-NADPH (10 mM in PB), 5 µL of either equine liver microsomes, human liver microsomes, equine S9 fractions or human S9 fractions. The tubes were incubated in a Thermomixer Compact from Eppendorf (Hamburg, Germany) at 37 °C and 300 rpm. The incubation was performed for 90 min, thereafter 10 µL UDPGA (10 mM in PB) was added and the incubation was continued for an additional 90 min. Several control samples were used, consisting of UDPGA blank (without addition of UDPGA) substrate blank (without addition of ACP-105), enzyme blank (without addition of microsomes/S9 fractions) and a positive control (with LGD-4033 instead of ACP-105). The incubation was terminated by the addition of 200 µL ice cold acetonitrile, vortexed and centrifuged at 4 °C, 11,500× *g* for 10 min in a Micro Star 17R from VWR (Radnor, PA, USA). An amount of 200 µL of the supernatant was transferred to a new vial and evaporated to dryness in a vacuum centrifuge. The samples were reconstituted in 100 µL aqueous formic acid (0.1%) and transferred to vials for analysis.

### 3.6. C. elegans Incubation

Fungal suspension (100 µL) was added to agar plates using an inoculating loop. The agar plates were placed in a Sanyo incubator MIR-253 (Osaka, Japan) together with agar plates without fungal suspension as control samples. They were incubated at 27 °C and growth was visible after two days. After five days the fungal mycelium was transferred to a container with 75.0 mL sodium chloride solution (0.86–0.90%) and stored at 5 °C. Sabouraud dextrose broth was produced by dissolving 30.0 g in water and autoclaved at 121 °C for 15 min. An amount of 6.0 mL broth was added to test tubes containing 0.5 mg of ACP-105 and 833 µL of fungal suspension was added. The test tubes were incubated at 27 °C for five days, control samples without ACP-105 and without fungal suspension were also incubated. The incubation was terminated by addition of 4.0 mL ice-cold acetonitrile. From the incubate, 500 µL was transferred to an Eppendorf tube and centrifuged at 11,500× *g* for 10 min, the supernatant was transferred to a vial and diluted 1:10 with aqueous formic acid (0.1%) prior to analysis.

### 3.7. Analysis

The chromatographic separation was performed using a Vanquish UHPLC^+^ focused binary pump F and autosampler Vanquish Split Sampler FT from Thermo Fisher Scientific (Waltham, MA, USA) at 40 °C using an Acquity UPLC^®^ HSS T3 column (2.1 × 100 mm; particle size 1.8 µm) with an HSS T3 guard column (2.1 × 5 mm; particle size 1.8 µm) from Waters (Milford, MA, USA). Mobile phase A consisted of 0.1% formic acid in water and mobile phase B consisted of 0.1% formic acid in acetonitrile. The flow rate was set to 0.45 mL·min^−1^ and the initial condition of 3% of mobile phase B was held for 1 min, before starting a linear gradient from 3–97% of mobile phase B over 16 min and thereafter isocratically holding at 97% for 4 min. The composition was thereafter changed to 3% of mobile phase B and held for 3 min for re-equilibration. The injection volume was 5 µL.

The UHPLC system was hyphenated to a Q Exactive^TM^ Orbitrap^TM^ benchtop mass spectrometer equipped with a heated electrospray ionization probe (HESI-II), all from Thermo Fisher Scientific (Waltham, MA, USA) used through the software TraceFinder^TM^ 5.1. The spray voltage vas 3.5 kV in positive ionization, and −3.0 kV in negative ionization. The capillary temperature was 320 °C and the aux gas heater temperature 400 °C. The sheath gas was 50, auxiliary gas 10, S-lens RF level 60; all in arbitrary units. Nitrogen was used as collision gas.

The mass calibration was performed using a calibration solution from the manufacturer containing caffeine, MRFA and Ultramark 1621. Data analysis was performed using the software FreeStyle^TM^ 1.7 from Thermo Fisher Scientific. The system was operated at a resolution of 70,000 at *m*/*z* 200 at full width at half maximum (FWHM), in full scan mode (*m*/*z* 120-1000). Full scan MS, full scan MS/ddMS2 and data independent MS/MS analysis were performed. The normalized collision energy for MS/MS analysis varied depending on the metabolite, but general settings were 10, 35 and 70 %. The isolation window for MS/MS experiments was ± 0.2 *m*/*z*. The metabolite search was performed by utilizing the theoretical metabolic transformations such as hydroxylations, glucuronidations and sulfatations and the theoretical chemical compositions. All results were compared with those of relevant blank reference samples and MS/MS spectra were interpreted and compared with that of the parent compound. The search was performed in both positive and negative mode. The sensitivity and retention time stability were monitored by injections of the independent reference compound RAD-140 at the beginning and end of each batch. Each biological sample was analyzed a minimum of five times.

## 4. Conclusions

ACP-105 was excreted to a minor extent in unchanged form and is very prone to metabolic transformations in the horse. A total of 21 metabolites were tentatively identified, out of which five major metabolites were selected for detailed characterization. These were the monohydroxylated metabolite M1a and the corresponding monohydroxylated glucuronide M8a, the monohydroxylated metabolite M1c and the corresponding monohydroxylated glucuronide M8d together with the dihydroxylated metabolite with a net loss of two hydrogens, M5b. Seven novel phase II metabolites of ACP-105 were tentatively identified as glucuronides in this study.

This study shows the importance and the usefulness of comparing with relevant in vitro models. The results showed that with the described method, equine microsomes can be used as reference material in doping analysis since they produce the major metabolites M1a and M1c. *C. elegans* also produces the metabolite M1a. The use of the metabolites M1a and M1c as analytical targets in hydrolyzed urine and metabolite M5b as target in plasma prolongs the detection time and thereby increases the possibility to detect illicit use of ACP-105.

## Data Availability

The data presented in this study are available within the article and supplementary material.

## Supplementary Materials

The following are available online at https://www.mdpi.com/2218-1989/11/2/85/s1, Figure S1: Mass spectrum and suggested fragments for all tentatively identified metabolites (M1a–M9b).

## Appendix A

**Table A1:** The metabolites from ACP-105 tentatively identified in plasma, urine and hydrolyzed urine (H. urine) which represents urine treated with β-glucuronidase. The theoretical *m*/*z* (Th. *m*/*z*) is based on the elemental composition and the experimental *m*/*z* (Ex. *m*/*z*) is from analysis with positive electrospray ionization (ESI). The column Detectability after administration indicates for how long the analyte was detectable in the different matrices after administration and represents horse A and B in the format of A/B h. The samples were taken 0–96 h from the time of administration. A total of 21 metabolites from ACP-105 were tentatively identified, labeled M1a–M9b consisting of hydroxylations, loss of two hydrogens, glucuronidations and combinations of these metabolic transformations.

Metabolite	Elemental Composition [M+H]^+^	r_t_ (min)	Th. *m*/*z* (T) Ex. *m*/*z* (E) ppm	Fragment *m*/*z* (ppm)	Neutral Loss	Detectability after Administration	
**ACP-105**	C_16_H_20_ClN_2_O^+^	11.59	T: 291.1259 E: 291.1262 −1.2 ppm	273.1149 (1.4) 233.0845 (−2.2) 193.0532 (−2.6) 179.0373 (−1.4) 167.0373 (−1.5) 125.0960 (0.7) 107.0856 (−0.7)	H_2_O C_3_H_6_O C_6_H_10_O C_7_H_12_O C_8_H_12_O C_8_H_7_ClN_2_ C_8_H_7_ClN_2_, H_2_O	Plasma: Urine: H. urine:	24/12 h 24/24 h 48/48 h
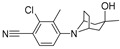
**M1a**	Monohydroxylation C_16_H_20_ClN_2_O_2_^+^	8.65	T: 307.1208 E: 307.1209 −0.5 ppm	289.1105 (−1.0) 231.0685 (−0.7) 195.0918 (−0.7) 189.0215 (−0.6) 177.0215 (−0.6) 142.0527 (−1.4) 107.0856 (−0.7)	H_2_O C_3_H_8_O_2_ C_3_H_8_O_2_, HCl C_6_H_14_O_2_ C_7_H_14_O_2_ C_7_H_14_O_2_, Cl^•^ C_8_H_5_ClN_2_, 2xH_2_O	Plasma: Urine: H. urine:	36/24h - 96/96 h
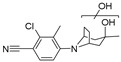
**M1b**	Monohydroxylation C_16_H_20_ClN_2_O_2_^+^	9.00	T: 307.1208 E: 307.1210 −0.8 ppm	289.1104 (−0.7) 271.0999 (−1.0) 233.0477 (−0.4) 193.0529 (−1.1) 179.0371 (−0.3) 142.0527 (−1.4) 105.0696 (2.6)	H_2_O 2xH_2_O C_4_H_10_O C_6_H_10_O_2_ C_7_H_12_O_2_ C_7_H_14_O_2_, Cl^•^ C_8_H_7_ClN_2_, 2xH_2_O	Plasma: Urine: H. urine:	- - 48/72 h
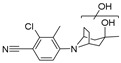
**M1c**	Monohydroxylation C_16_H_20_ClN_2_O_2_^+^	9.14	T: 307.1208 E: 307.1209 −0.5 ppm	289.1104 (−0.7) 271.0999 (−1.0) 233.0842 (−0.9) 193.0529 (−1.1) 179.0371 (−0.3) 167.0372 (−0.9) 123.0805 (−0.5) 105.0700 (−1.2)	H_2_O 2xH_2_O C_3_H_6_O_2_ C_6_H_10_O_2_ C_7_H_12_O_2_ C_8_H_12_O_2_ C_8_H_7_ClN_2_, H_2_O C_8_H_7_ClN_2_, 2xH_2_O	Plasma: Urine: H. urine:	36/24 h - 96/96 h
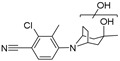
**M2a**	Dihydroxylation C_16_H_20_ClN_2_O_3_^+^	7.03	T: 323.1157 E: 323.1159 −0.7 ppm	305.1053 (−0.6) 231.0685 (−0.7) 195.0918 (−0.7) 189.0215 (−0.6) 177.0215 (−0.6) 142.0527 (−1.4)	H_2_O C_3_H_8_O_3_ C_3_H_8_O_3_, HCl C_6_H_14_O_3_ C_7_H_14_O_3_ C_7_H_14_O_3_, Cl^•^	Plasma: Urine: H. urine:	36/48 h -/7 h 48/24 h
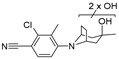
**M2b**	Dihydroxylation C_16_H_20_ClN_2_O_3_^+^	7.63	T: 323.1157 E: 323.1161 −1.3 ppm	305.1053 (−0.6) 287.0948 (−0.9) 247.0635 (−1.0) 229.0529 (−0.9) 194.0840 (−1.0) 179.0604 (0.0) 142.0527 (−1.4)	H_2_O 2xH_2_O C_3_H_8_O_2_ C_3_H_10_O_3_ C_3_H_10_O_3_, Cl^•^ C_4_H_12_O_3_, HCl C_7_H_14_O_3_, Cl^•^	Plasma: Urine: H. urine:	- - 72/48 h
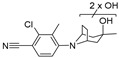
**M3a ^1^**	Loss of 2H C_16_H_18_ClN_2_O^+^	10.11	T: 289.1102 E: 289.1106 −1.4 ppm	271.1000 (−1.3) 243.0685 (−0.7) 233.0477 (−0.4) 179.0371 (−0.3) 177.0214 (0.0) 142.0526 (−0.7) 111.0805 (−0.6)	H_2_O C_2_H_6_O C_4_H_8_ C_7_H_10_O C_7_H_12_O C_7_H_12_O, Cl^•^ C_9_H_7_ClN_2_	Plasma: Urine: H. urine:	- - 24/24 h
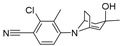
**M3b ^1,2^**	Loss of 2H C_16_H_18_ClN_2_O^+^	11.56	T: 289.1102 E: 289.1108 −2.1 ppm	271.1000 (−1.3) 229.0523 (1.7) 203.0370 (0.2) 183.0321 (−0.8) 165.0215 (−0.7)	H_2_O C_3_H_8_O C_5_H_10_O C_8_H_10_ C_8_H_12_O	Plasma: Urine: H. urine:	- 24/7 h 48/48 h
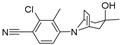
**M4a ^1^**	Monohydroxylation + loss of 2H C_16_H_18_ClN_2_O_2_^+^	8.88	T: 305.1051 E: 305.1054 −0.9 ppm	287.0949 (−1.2) 245.0842 (−0.9) 229.0529 (−0.9) 194.0840 (−1.0) 179.0605 (−0.7) 142.0527 (−1.4) 123.0805 (−0.5)	H_2_O C_2_H_4_O_2_ C_3_H_8_O_2_ C_3_H_8_O_2_, Cl^•^ C_4_H_10_O_2_, HCl C_7_H_12_O_2_, Cl^•^ C_8_H_5_ClN_2,_ H_2_O	Plasma: Urine: H. urine:	- -/7 h 48/48 h
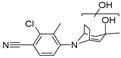
**M4b ^1^**	Monohydroxylation + loss of 2H C_16_H_18_ClN_2_O_2_^+^	9.83	T: 305.1051 E: 305.1053 −0.6 ppm	223.0270 (−0.6) 177.0215 (−0.6) 142.0527 (−1.4) 111.0441 (−0.5)	C_6_H_10_ C_7_H_12_O_2_ C_7_H_12_O_2_, Cl^•^ C_10_H_11_ClN_2_	Plasma: Urine: H. urine:	- - 72/48 h
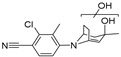
**M5a ^1,2^**	Dihydroxylation + loss of 2H C_16_H_18_ClN_2_O_3_^+^	9.17	T: 321.1000 E: 321.1005 −1.6 ppm	303.0897 (−0.8) 285.0793 (−1.4) 229.0530 (−1.3) 179.0375 (−2.6)	H_2_O 2xH_2_O C_3_H_8_O_3_ C_7_H_10_O_3_	Plasma: Urine: H. urine:	- 24/7 h 24/24 h
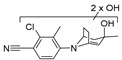
**M5b ^1^**	Dihydroxylation + loss of 2H C_16_H_18_ClN_2_O_3_^+^	9.57	T: 321.1000 E: 321.1003 −0.9 ppm	303.0896 (−0.5) 285.0792 (−1.0) 275.0949 (−1.3) 229.0530 (−1.3) 217.0529 (−1.0) 193.0528 (−0.6) 177.0215 (−0.6) 167.0372 (−0.9) 142.0527 (−1.4)	H_2_O 2xH_2_O C_1_H_2_O_2_ C_3_H_8_O_3_ C_4_H_8_O_3_ C_6_H_8_O_3_ C_7_H_12_O_3_ C_8_H_10_O_3_ C_7_H_12_O_3_, Cl^•^	Plasma: Urine: H. urine:	36/36 h 96/96 h 96/96 h
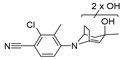
**M6a ^1^**	Trihydroxylation + loss of 2H C_16_H_18_ClN_2_O_4_^+^	7.21	T: 337.0950 E: 337.0953 −1.1 ppm	319.0847 (−1.0) 179.0372 (−0.9) 142.0527 (−1.4) 105.0336 (−1.1)	H_2_O C_7_H_10_O_4_ C_7_H_12_O_4_, Cl^•^ C_9_H_7_ClN_2,_ 3xH_2_O	Plasma: Urine: H. urine:	- 72/24 h 72/48 h
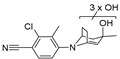
**M6b ^1^**	Trihydroxylation + loss of 2H C_16_H_18_ClN_2_O_4_^+^	7.47	T: 337.0950 E: 337.0951 −0.5 ppm	319.0845 (−0.4) 231.0686 (−1.1) 195.0918 (−0.7) 189.0215 (−0.6) 177.0215 (−0.6) 142.0527 (−1.4)	H_2_O C_3_H_6_O_4_ C_3_H_6_O_4_, HCl C_6_H_12_O_4_ C_7_H_12_O_4_ C_7_H_12_O_4_, Cl^•^	Plasma: Urine: H. urine:	-/6 h 72/24 h 72/72 h
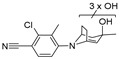
**M6c ^1,2^**	Trihydroxylation + loss of 2H C_16_H_18_ClN_2_O_4_^+^	9.04	T: 337.0950 E: 337.0955 −1.7 ppm	319.0847 (−1.0) 301.0742 (−1.3)	H_2_O 2xH_2_O	Plasma: Urine: H. urine:	- 24/24 h 6/24 h
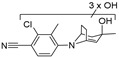
**M7**	Glucuronidation C_22_H_28_ClN_2_O_7_^+^	8.95	T: 467.1580 E: 467.1580 0.0 ppm	291.1259 (0.0) 273.1155 (−0.8) 233.0841 (−0.5) 193.0529 (−1.1) 179.0371 (−0.3) 167.0372 (−0.9) 142.0527 (−1.4) 107.0856 (−0.7)	C_6_H_8_O_6_ H_2_O, C_6_H_8_O_6_ C_3_H_6_O, C_6_H_8_O_6_ C_6_H_10_O, C_6_H_8_O_6_ C_7_H_12_O, C_6_H_8_O_6_ C_8_H_12_O, C_6_H_8_O_6_ C_7_H_14_O, Cl^•^, C_6_H_8_O_6_ C_8_H_5_ClN_2_, 2xH_2_O, C_6_H_8_O_6_	Plasma: Urine: H. urine:	-/6 h 24/24 h -

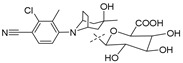

**M8a**	Monohydroxylation + glucuronidation C_22_H_28_ClN_2_O_8_^+^	7.13	T: 483.1529 E: 483.1531 −0.4 ppm	307.1210 (−0.8) 289.1104 (−0.7) 231.0685 (−0.7) 195.0919 (−1.2) 189.0216 (−1.1) 177.0215 (−0.6) 142.0527 (−1.4)	C_6_H_8_O_6_ H_2_O, C_6_H_8_O_6_ C_3_H_8_O_2_, C_6_H_8_O_6_ C_3_H_8_O_2_, HCl, C_6_H_8_O_6_ C_6_H_14_O_2_, C_6_H_8_O_6_ C_7_H_14_O_2_, C_6_H_8_O_6_ C_7_H_14_O_2_, Cl^•^, C_6_H_8_O_6_	Plasma: Urine: H. urine:	36/36 h 48/48 h 6/7 h

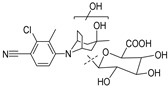

**M8b**	Monohydroxylation + glucuronidation C_22_H_28_ClN_2_O_8_^+^	7.61	T: 483.1529 E: 483.1527 0.4 ppm	465.1428 (−1.2) 289.1105 (−1.0) 271.0999 (−1.0) 217.0529 (−1.0) 179.0372 (−0.9) 123.0805 (−0.5)	H_2_O H_2_O, C_6_H_8_O_6_ 2xH_2_O, C_6_H_8_O_6_ C_4_H_10_O_2_, C_6_H_8_O_6_ C_7_H_12_O_2_, C_6_H_8_O_6_ C_8_H_7_ClN_2_, H_2_O, C_6_H_8_O_6_	Plasma: Urine: H. urine:	- 6/7 h -

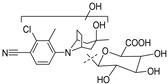

**M8c**	Monohydroxylation + glucuronidation C_22_H_28_ClN_2_O_8_^+^	7.85	T: 483.1529 E: 483.1524 1.0 ppm	307.1210 (−0.8) 289.1105 (−1.0) 271.1001 (−1.7) 233.0481 (−2.1) 193.0530 (−1.6) 179.0372 (−0.9) 142.0528 (−2.1)	C_6_H_8_O_6_ H_2_O, C_6_H_8_O_6_ 2xH_2_O, C_6_H_8_O_6_ C_4_H_10_O, C_6_H_8_O_6_ C_6_H_10_O_2_, C_6_H_8_O_6_ C_7_H_12_O_2_, C_6_H_8_O_6_ C_7_H_14_O_2_, Cl^•^, C_6_H_8_O_6_	Plasma: Urine: H. urine:	- 24/24 h -

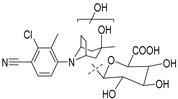

**M8d**	Monohydroxylation + glucuronidation C_22_H_28_ClN_2_O_8_^+^	8.00	T: 483.1529 E: 483.1530 −0.2 ppm	465.1425 (−0.5) 307.1209 (−0.5) 289.1104 (−0.7) 271.0999 (−1.0) 233.0841 (−0.5) 193.0528 (−0.6) 179.0371 (−0.3) 167.0372 (−0.9) 123.0805 (−0.5)	H_2_O C_6_H_8_O_6_ H_2_O, C_6_H_8_O_6_ 2xH_2_O, C_6_H_8_O_6_ C_3_H_6_O_2_, C_6_H_8_O_6_ C_6_H_10_O_2_, C_6_H_8_O_6_ C_7_H_12_O_2_, C_6_H_8_O_6_ C_8_H_12_O_2_, C_6_H_8_O_6_ C_8_H_7_ClN_2_, H_2_O, C_6_H_8_O_6_	Plasma: Urine: H. urine:	36/36 h 96/48 h -/7 h

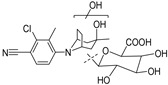

**M9a**	Dihydroxylation + glucuronidation C_22_H_28_ClN_2_O_9_^+^	6.21	T: 499.1478 E: 499.1470 1.6 ppm	323.1155 (0.5) 305.1053 (−0.6) 287.0947 (−0.5) 231.0685 (−0.7) 195.0919 (−1.2) 142.0527 (−1.4)	C_6_H_8_O_6_ H_2_O, C_6_H_8_O_6_ 2xH_2_O, C_6_H_8_O_6_ C_3_H_8_O_3_, C_6_H_8_O_6_ C_3_H_8_O_3_, HCl, C_6_H_8_O_6_ C_7_H_14_O_3_, Cl^•^, C_6_H_8_O_6_	Plasma: Urine: H. urine:	- 48/24 h 6/7 h

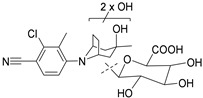

**M9b**	Dihydroxylation + glucuronidation C_22_H_28_ClN_2_O_9_^+^	6.52	T: 499.1478 E: 499.1478 0.0 ppm	481.1376 (−0.9) 323.1160 (−1.0) 305.1053 (−0.6) 287.0948 (−0.9) 247.0635 (−1.0) 231.0685 (−0.7) 195.0918 (−0.7) 142.0527 (−1.4)	H_2_O C_6_H_8_O_6_ H_2_O, C_6_H_8_O_6_ 2xH_2_O, C_6_H_8_O_6_ C_3_H_8_O_2_, C_6_H_8_O_6_ C_3_H_8_O_3_, C_6_H_8_O_6_ C_3_H_8_O_3_, HCl, C_6_H_8_O_6_ C_7_H_14_O_3_, Cl^•^, C_6_H_8_O_6_	Plasma: Urine: H. urine:	- 48/24 h -

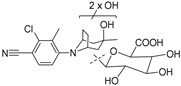

^1^ The position of the double bond within the aliphatic ring structure is not known. ^2^ Marked metabolites were tentatively identified at a low intensity and/or co-eluting with background analytes with an *m*/*z* ± 0.2 from the analyte of interest, causing the MS/MS spectrum to contain a combination of fragments from analytes giving a lower level of identification.
