# Supplementary material for: Investigation of Equine In Vivo and In Vitro Derived Metabolites of the Selective Androgen Receptor Modulator (SARM) ACP-105 for Improved Doping Control
Source: Metabolites. 2021 Feb 1;11(2):85. doi: 10.3390/metabo11020085 (PMC7912737; doi:10.3390/metabo11020085)

S1: Supplementary material

Structural elucidation and fragmentation of ACP-105 and tentatively identified metabolites

Metabolite	Metabolic transformation	Elemental composition $[M+H]^+$	Theoretical m/z
ACP-105	Administered substance	$C_{16}H_{20}ClN_2O^+$	291.1259
M1a-c	Monohydroxylation	$C_{16}H_{20}ClN_2O_2^+$	307.1208
M2a-b	Dihydroxylation	$C_{16}H_{20}ClN_2O_3^+$	323.1157
M3a-b	Loss of 2H	$C_{16}H_{18}ClN_2O^+$	289.1102
M4a-b	Monohydroxylation + Loss of 2H	$C_{16}H_{18}ClN_2O_2^+$	305.1051
M5a-b	Dihydroxylation + Loss of 2H	$C_{16}H_{18}ClN_2O_3^+$	321.1000
M6a-c	Trihydroxylation + Loss of 2H	$C_{16}H_{18}ClN_2O_4^+$	337.0950
M7	Glucuronidation	$C_{22}H_{28}ClN_2O_7^+$	467.1580
M8a-d	Monohydroxylation + Glucuronidation	$C_{22}H_{28}ClN_2O_8^+$	483.1529
M9a-b	Dihydroxylation + Glucuronidation	$C_{22}H_{28}ClN_2O_9^+$	499.1478

ACP-105

Administrated substance

Retention time: 11.59 min

m/z: 291.1262

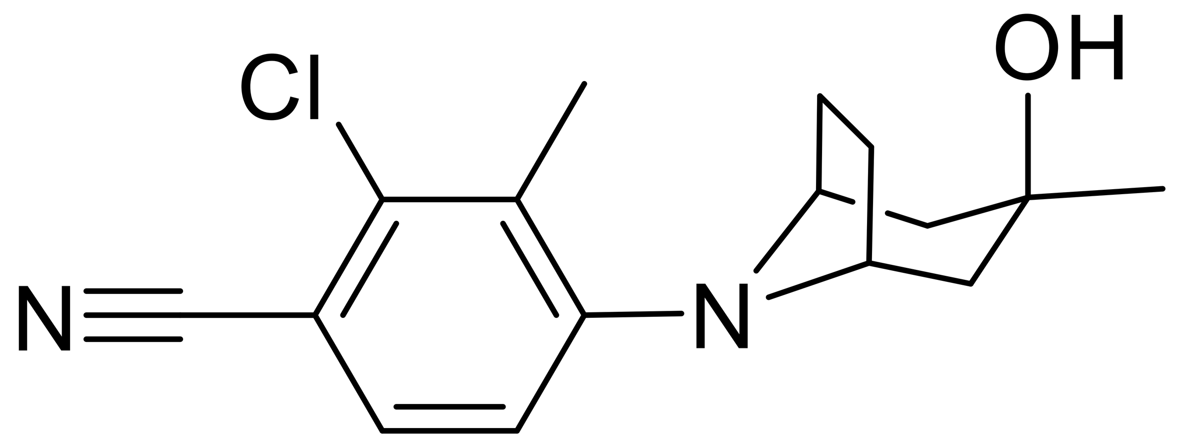

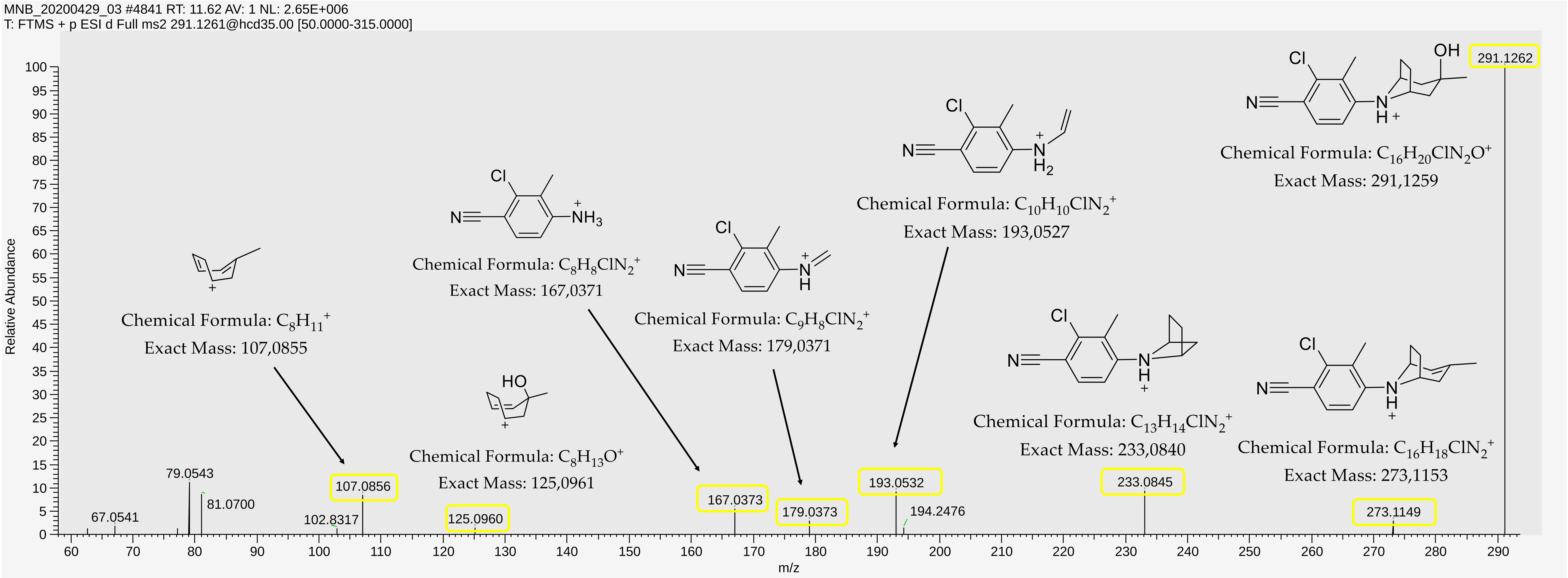

M1a

Monohydroxylation
Retention time: 8.65 min
m/z: 307.1209

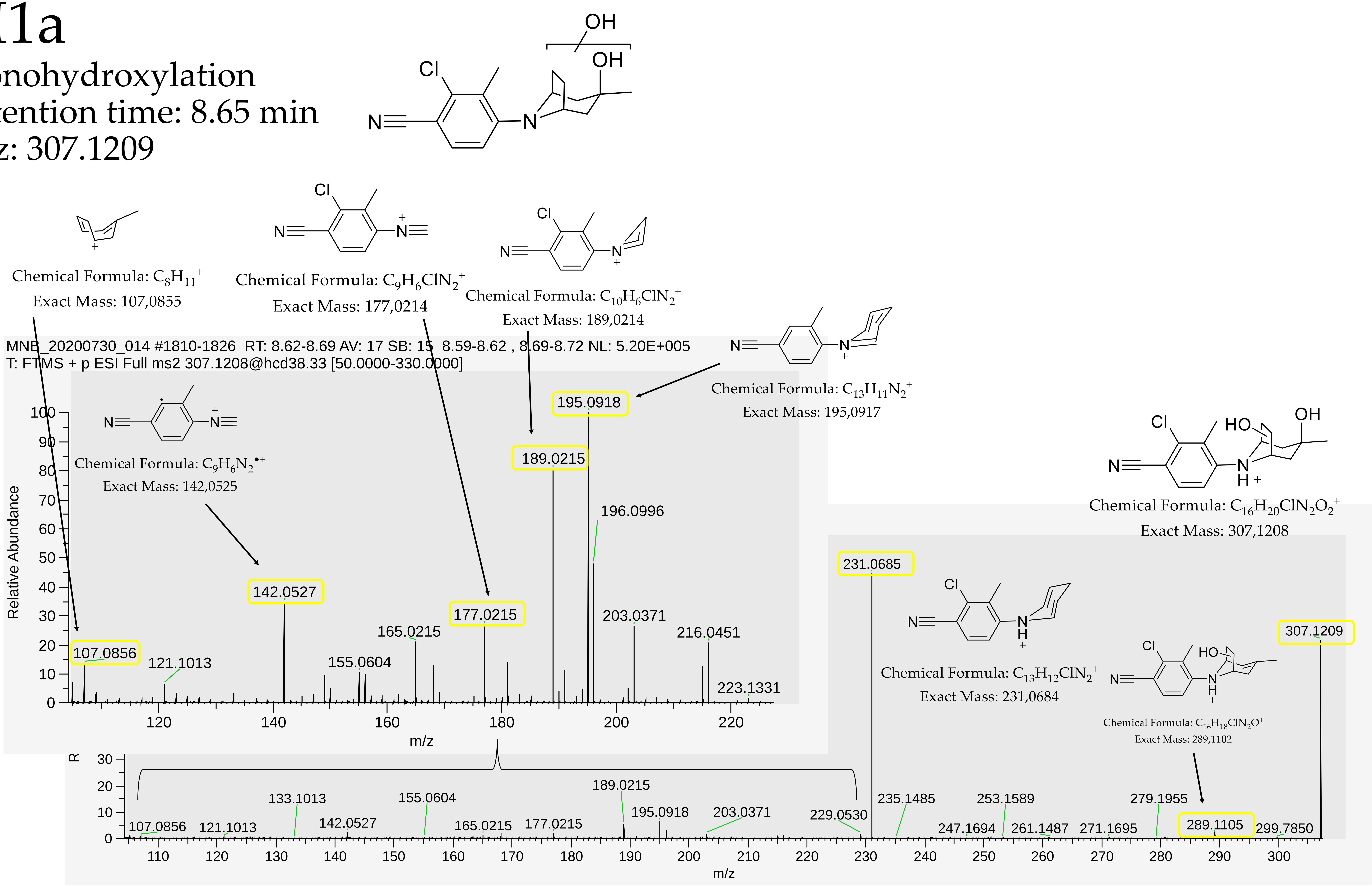

M1b

Monohydroxylation
Retention time: 9.00 min
m/z: 307.1210

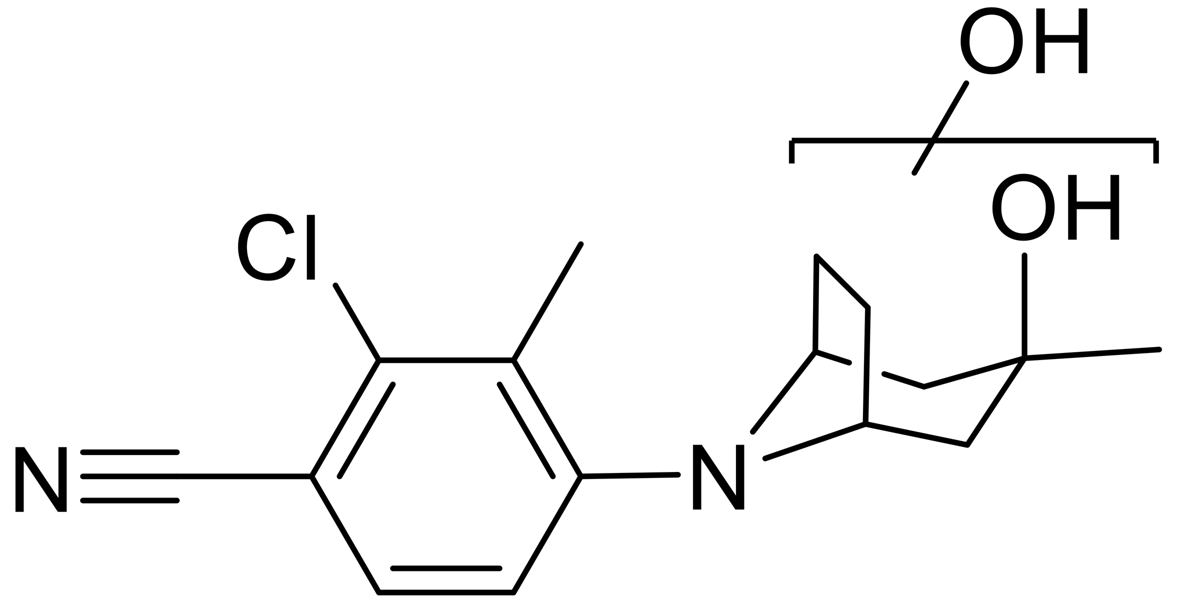

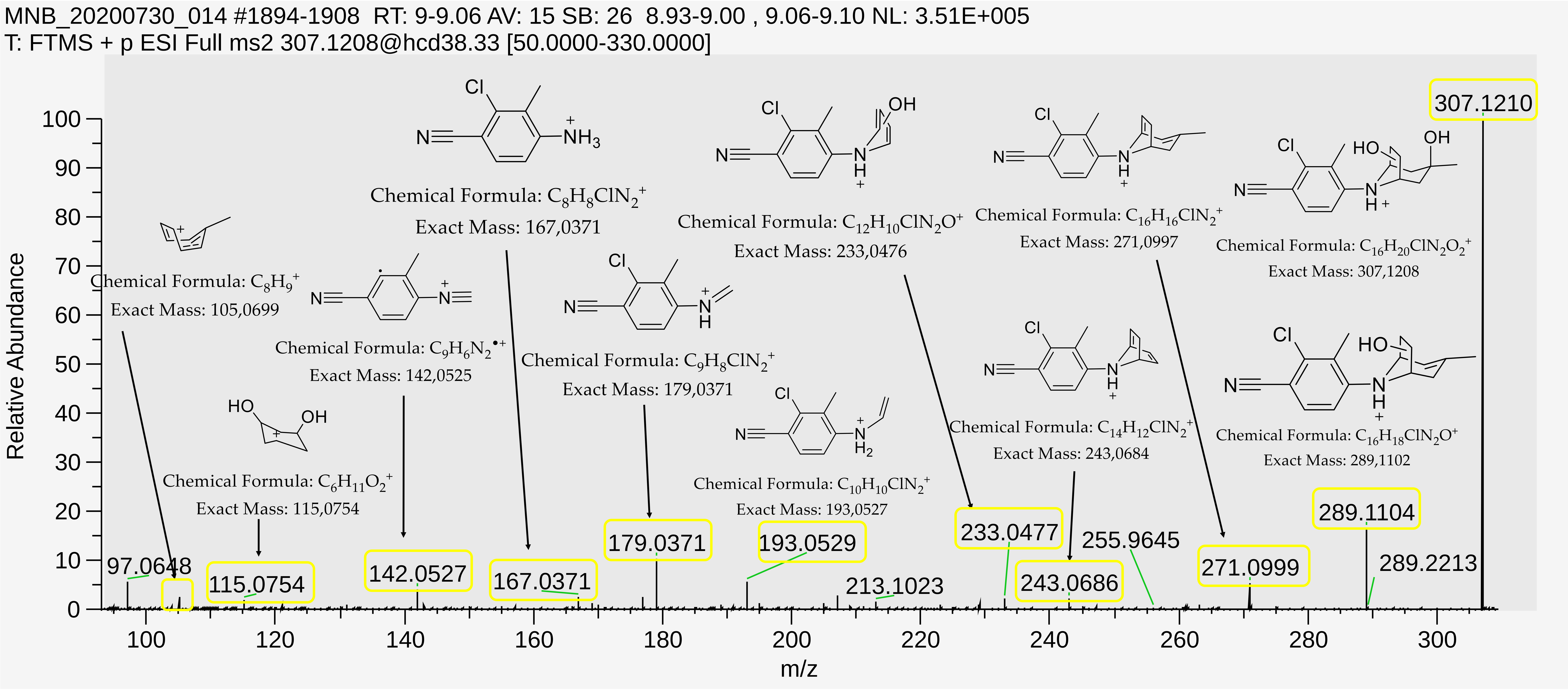

M1c

Monohydroxylation

Retention time: 9.14 min

m/z: 307.1209

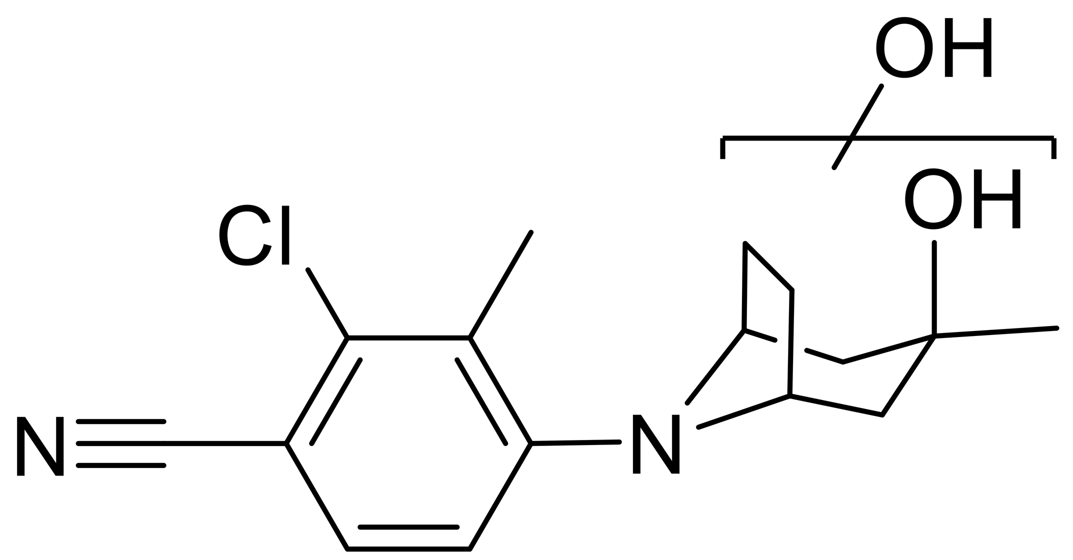

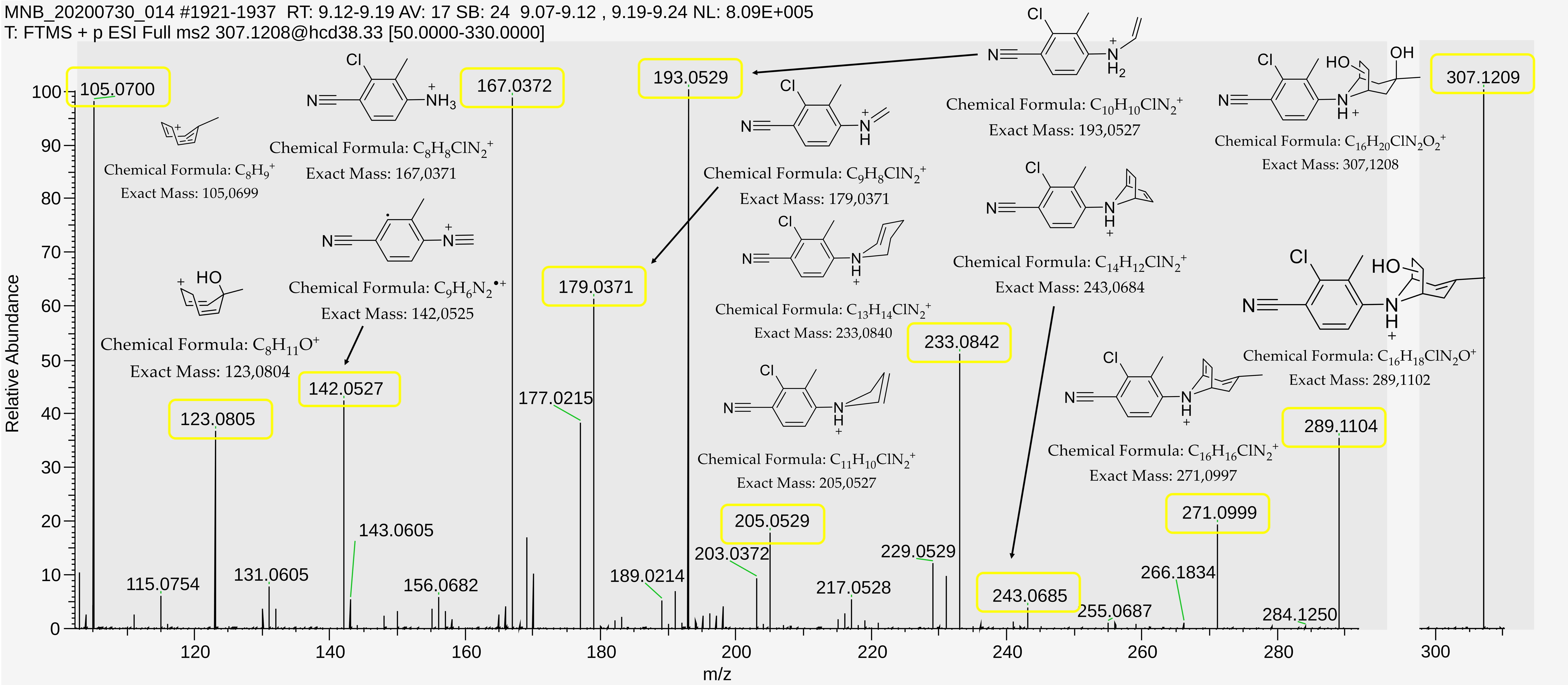

M2a

Dihydroxylation
Retention time: 7.03 min
m/z: 323.1159

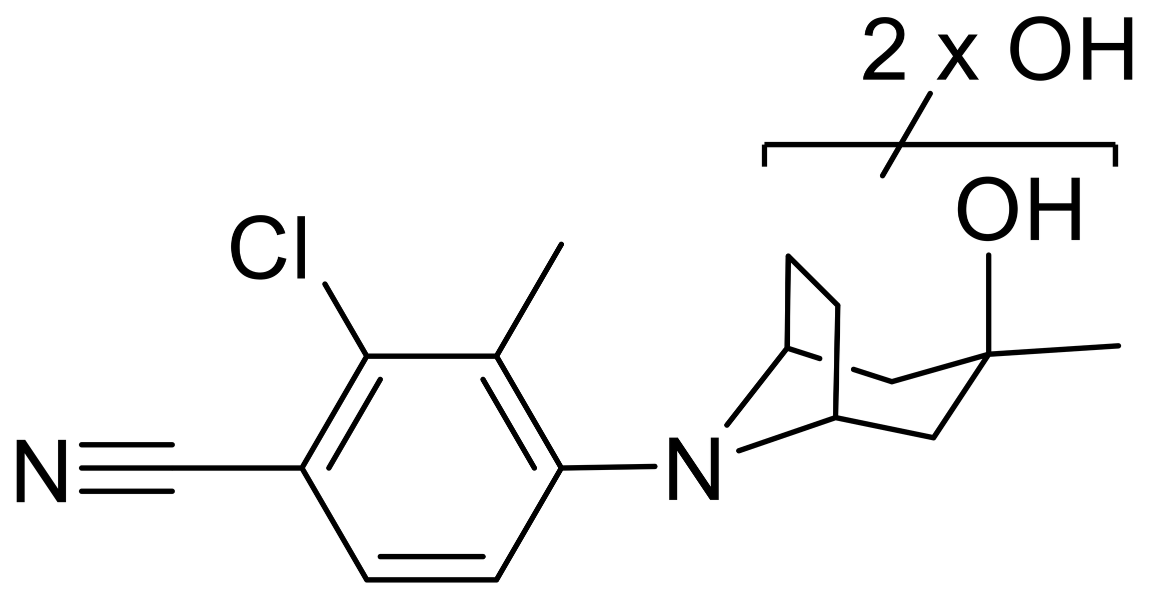

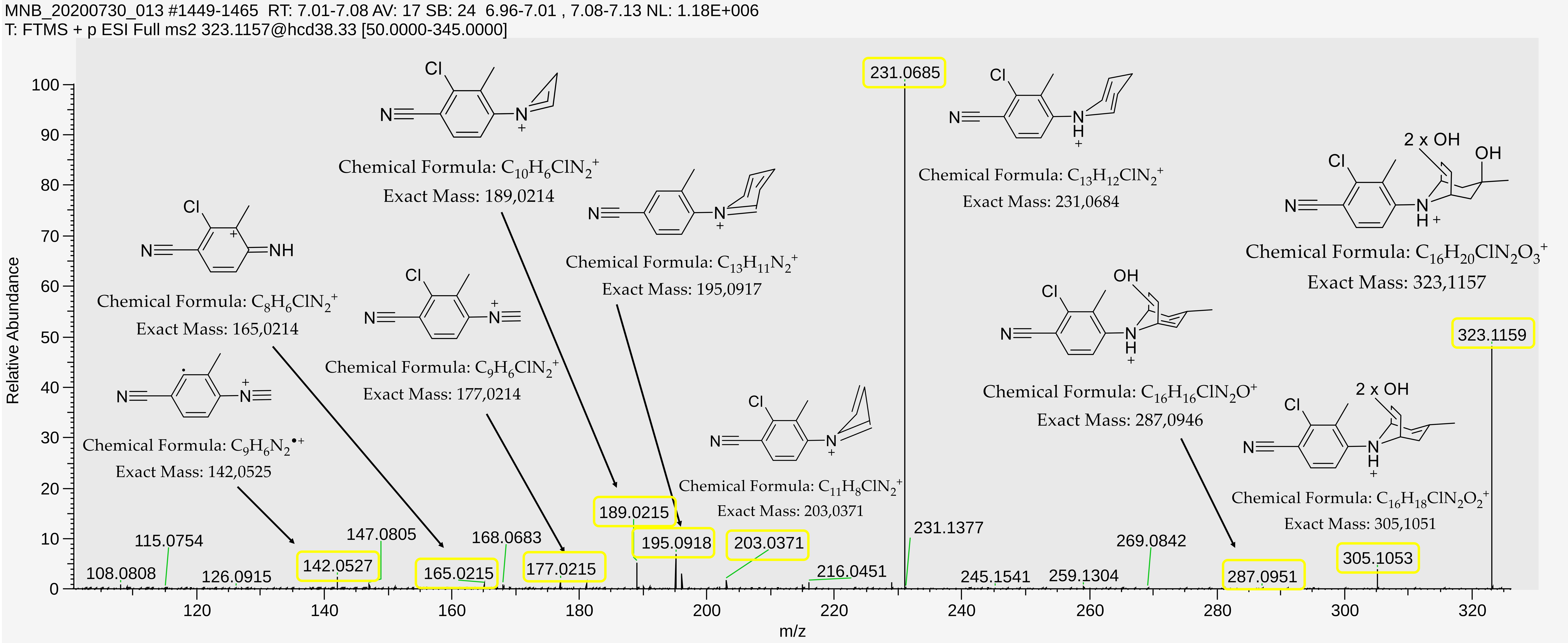

M2b

Dihydroxylation

Retention time: 7.63 min

m/z: 323.1161

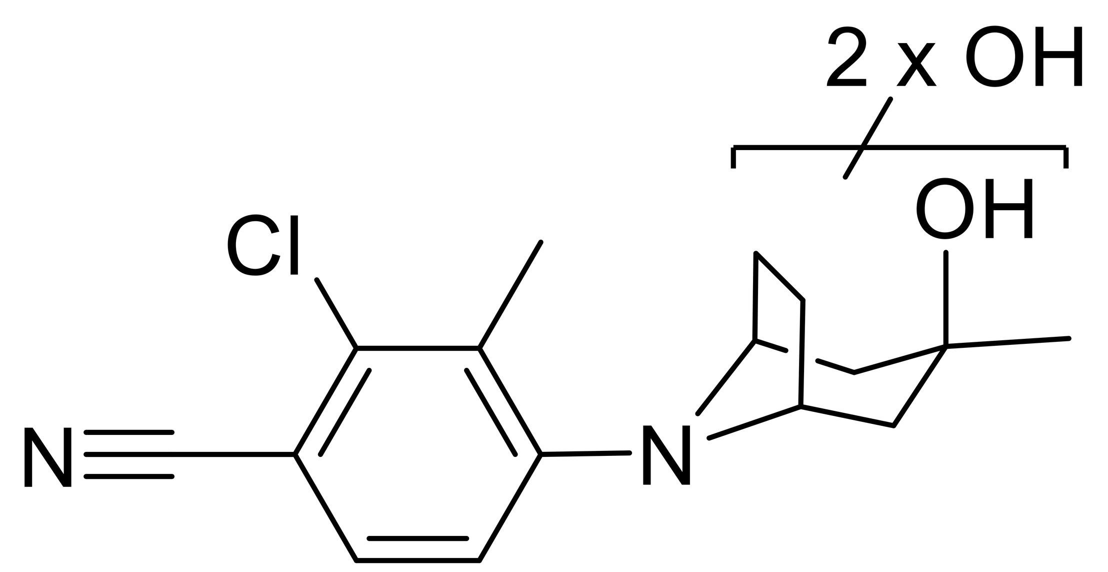

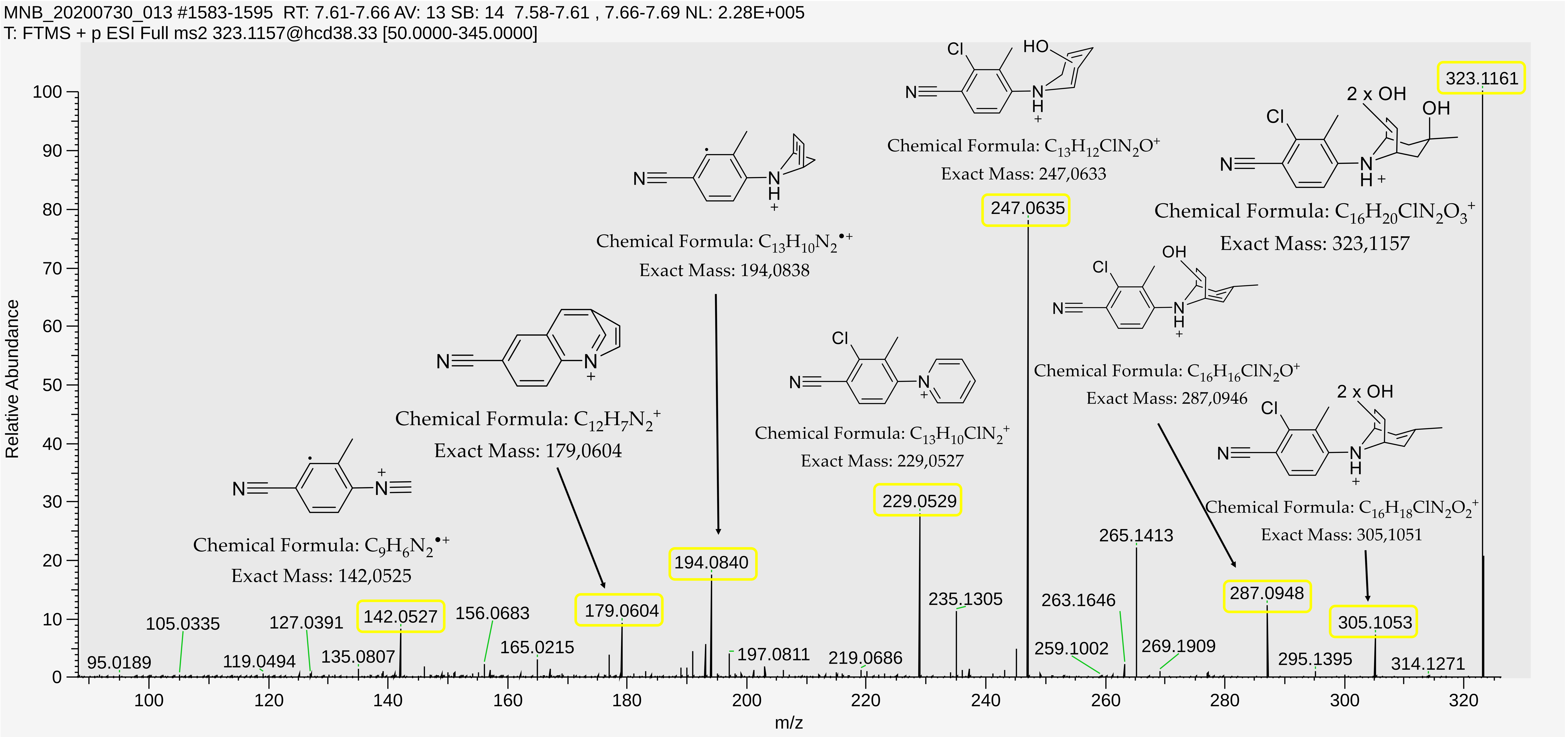

M3a

Loss of 2H
Retention time: 10.11 min
m/z: 289.1106

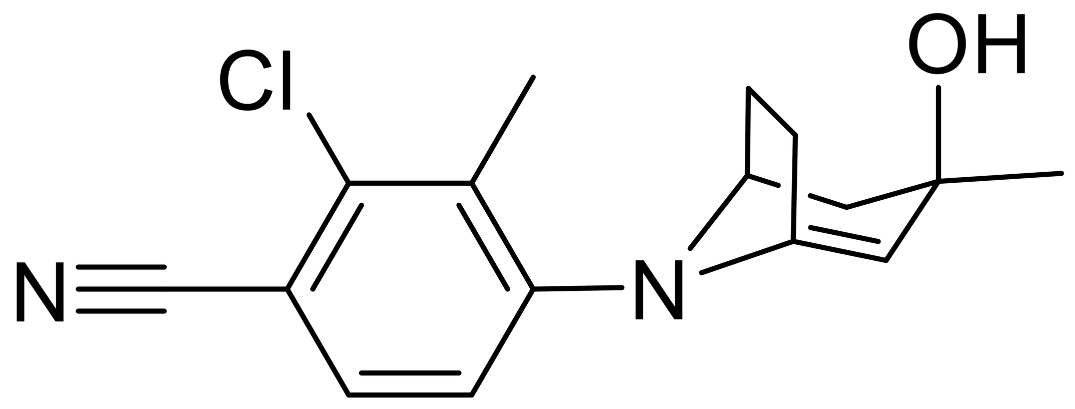

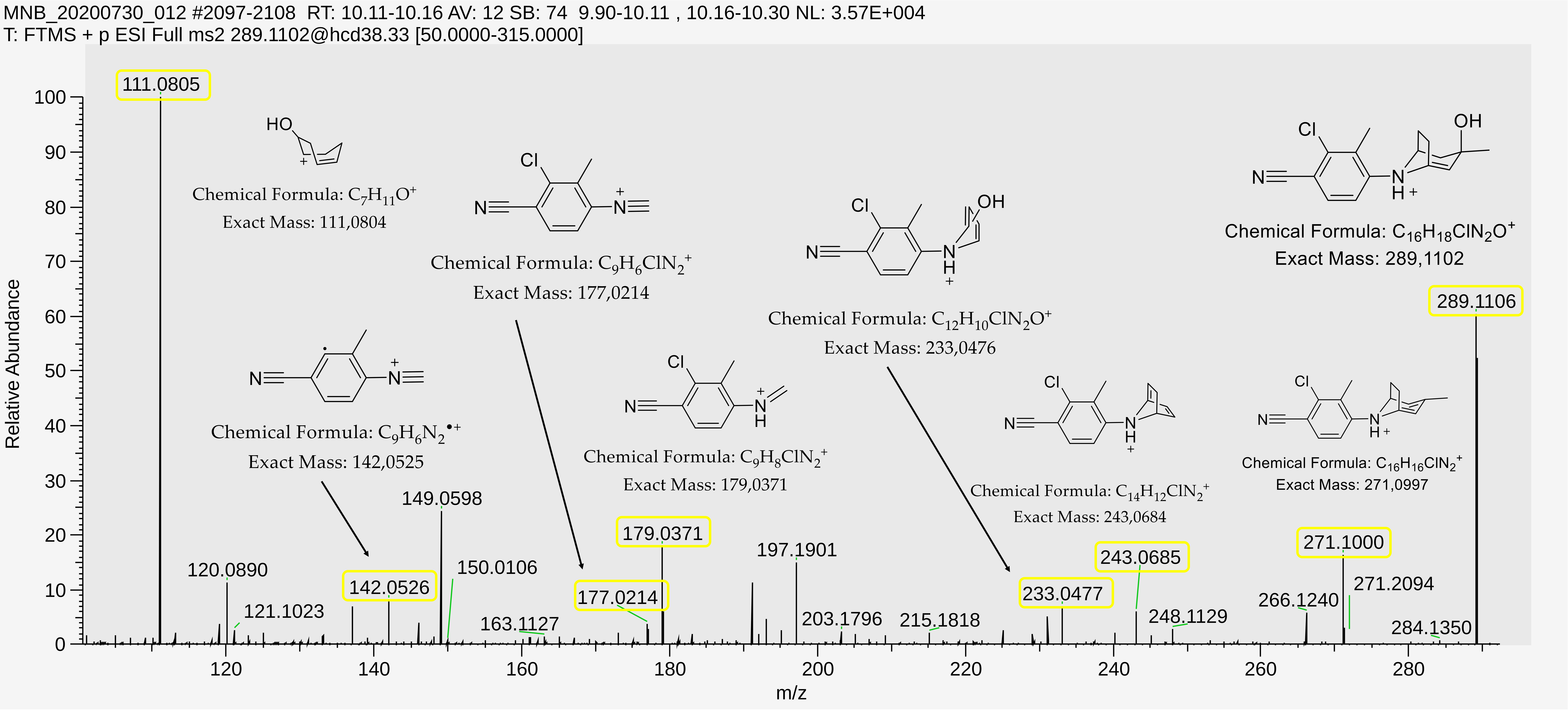

M3b

Loss of 2H
Retention time: 11.56 min
m/z: 289.1108

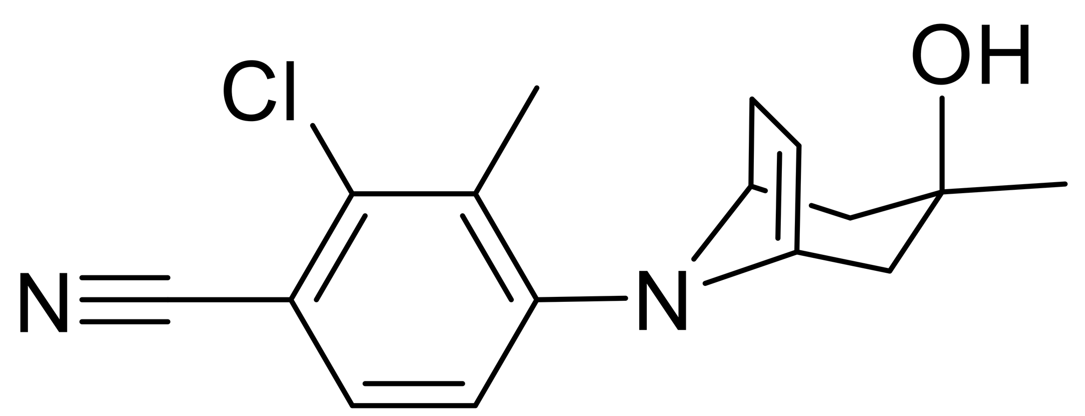

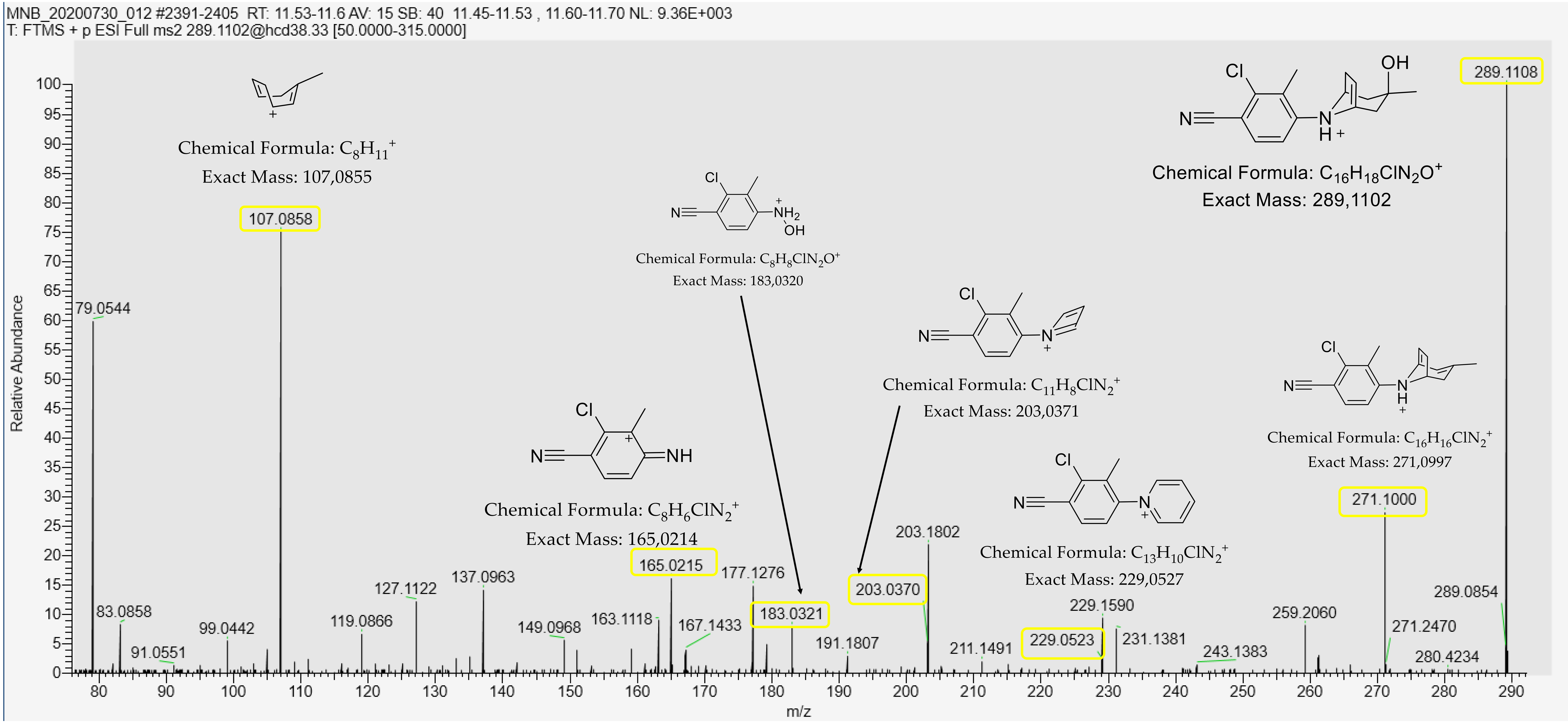

M4a

Monohydroxylation + loss of 2H
Retention time: 8.88 min
m/z: 305.1054

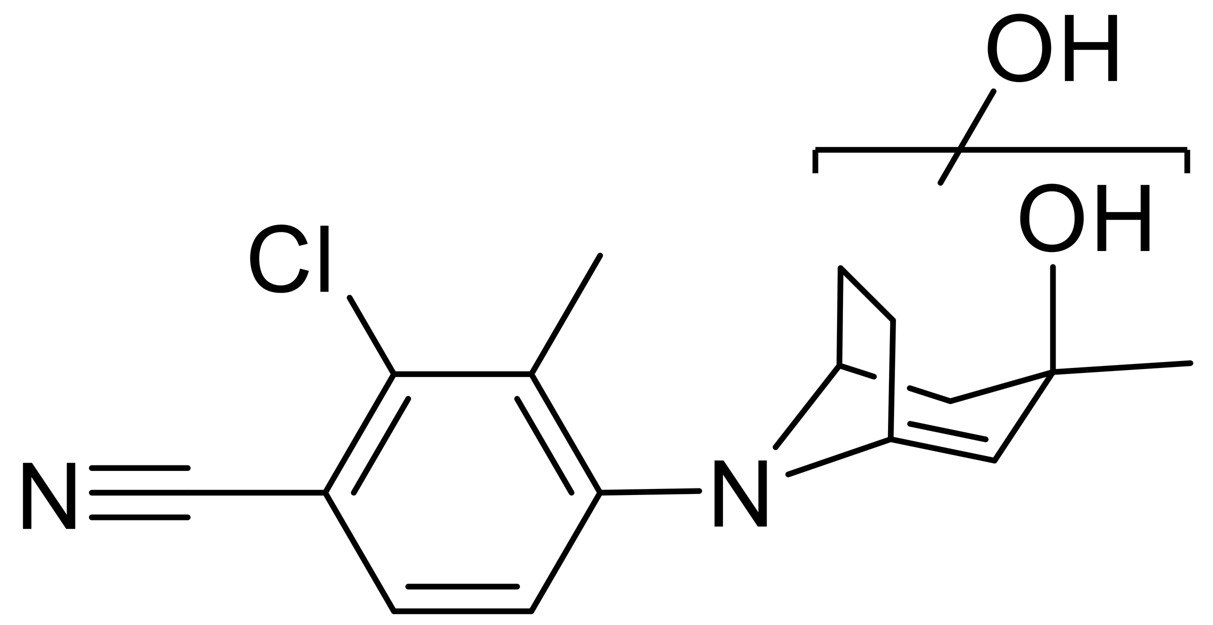

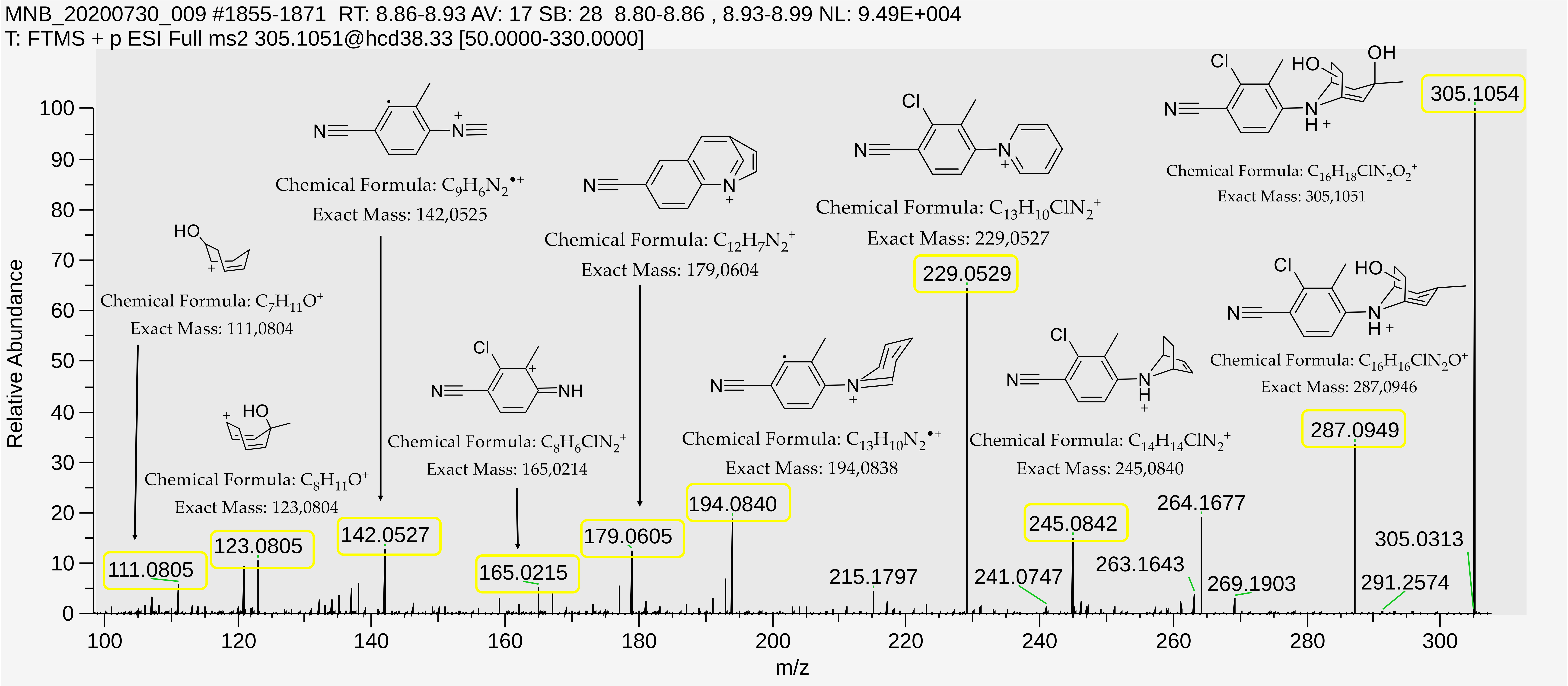

M4b

Monohydroxylation + loss of 2H
Retention time: 9.83 min
m/z: 305.1053

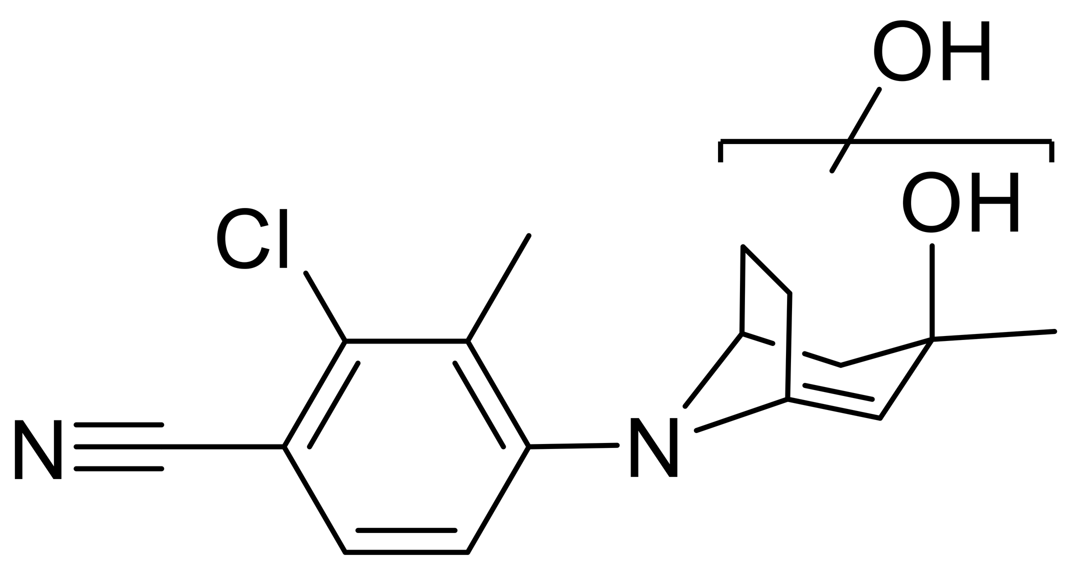

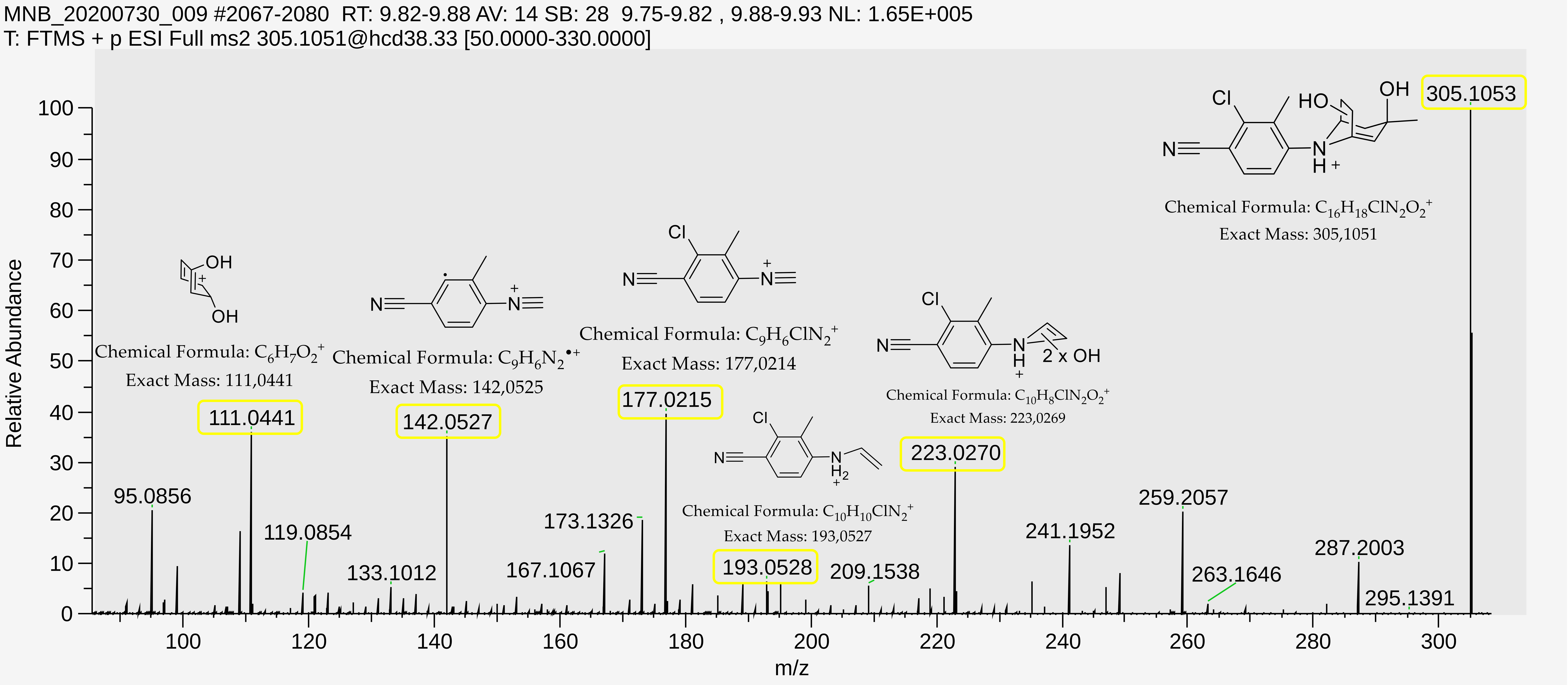

M5a

Dihydroxylation + loss of 2H
Retention time: 9.17 min
m/z: 321.1005

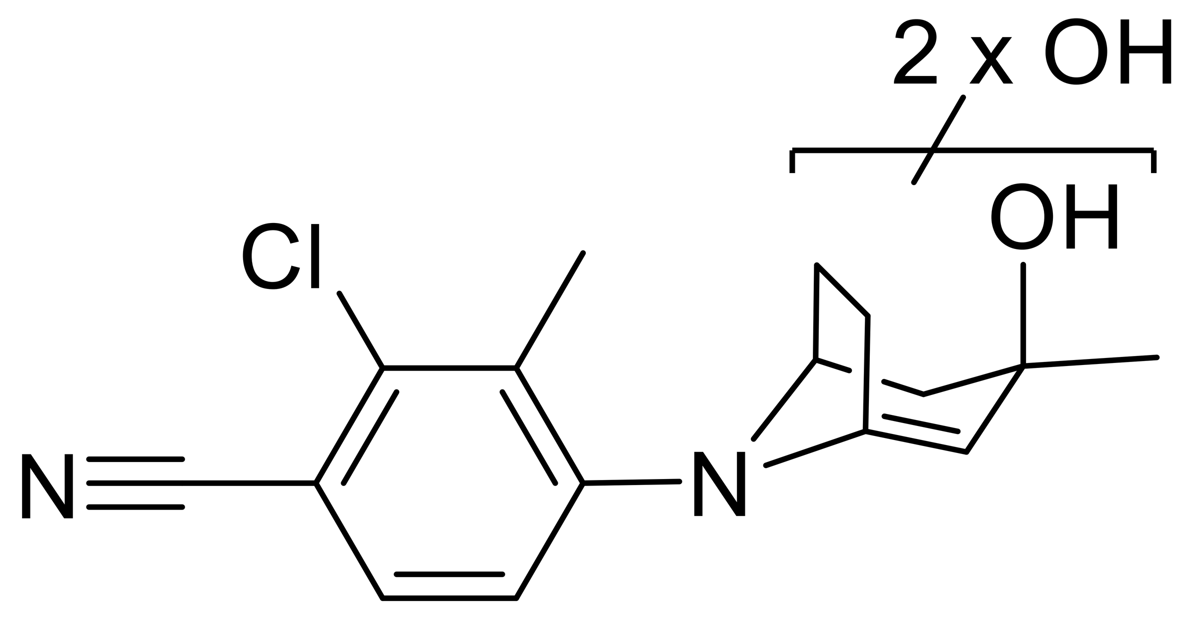

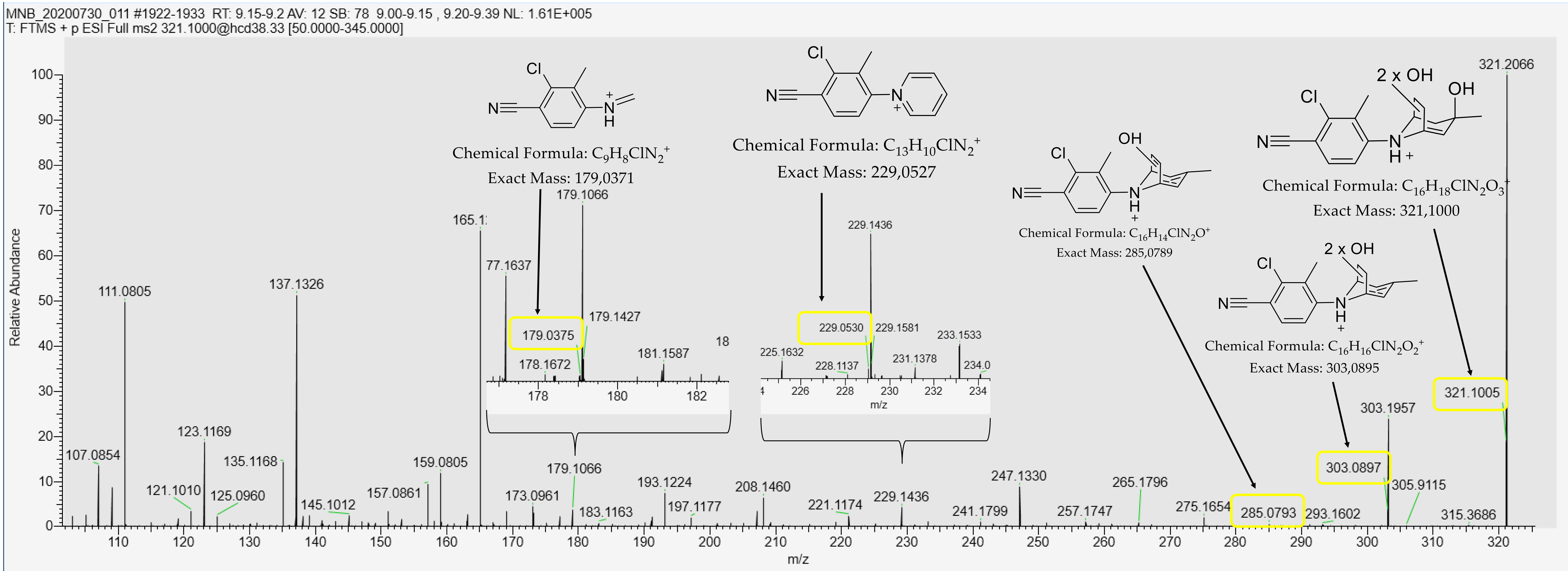

M5b

Dihydroxylation + loss of 2H
Retention time: 9.57 min
m/z: 321.1003

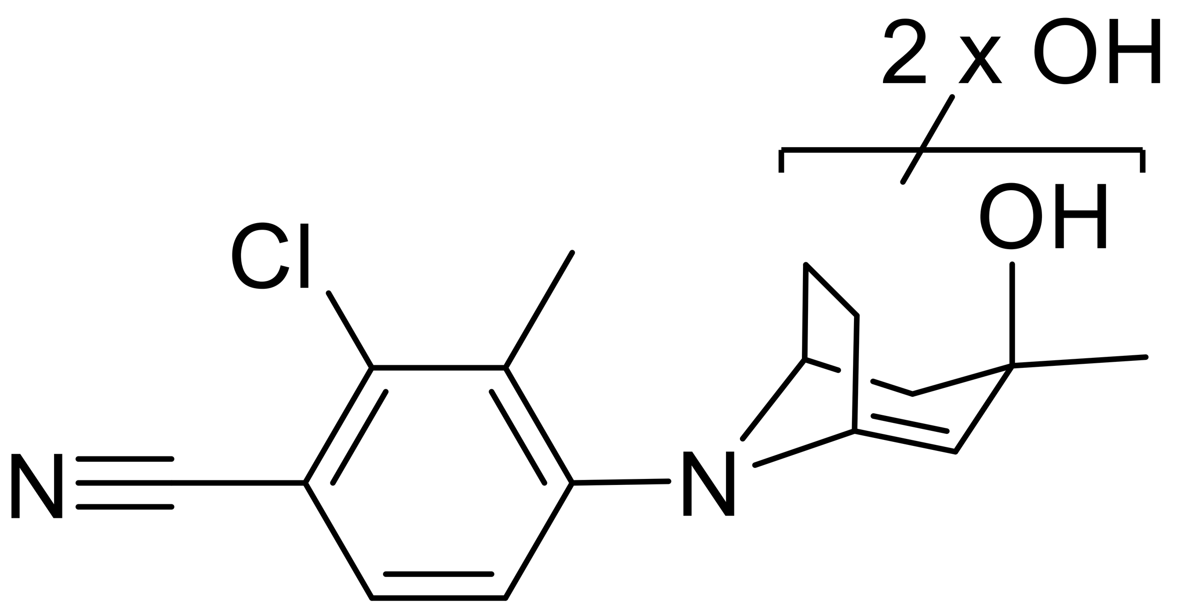

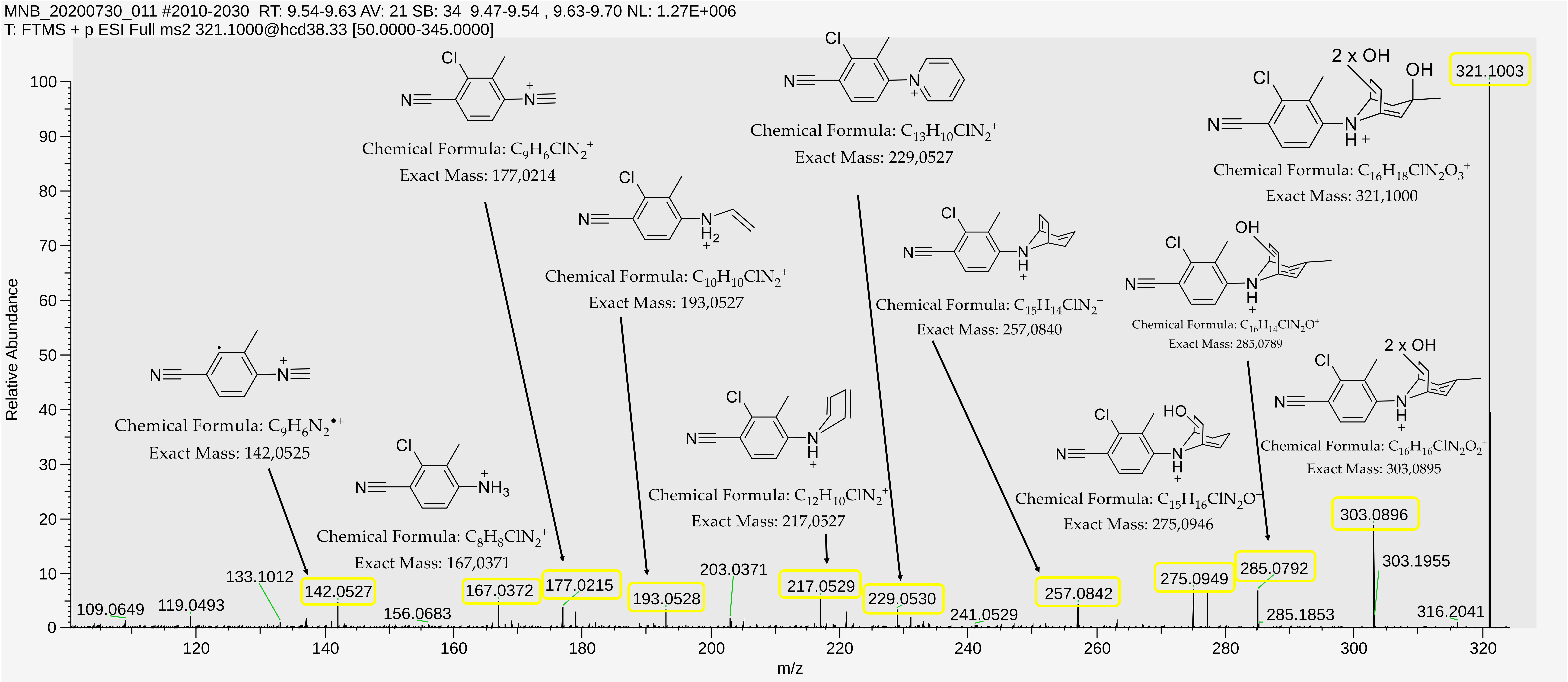

M6a

Trihydroxylation + loss of 2H
Retention time: 7.21 min
m/z: 337.0953

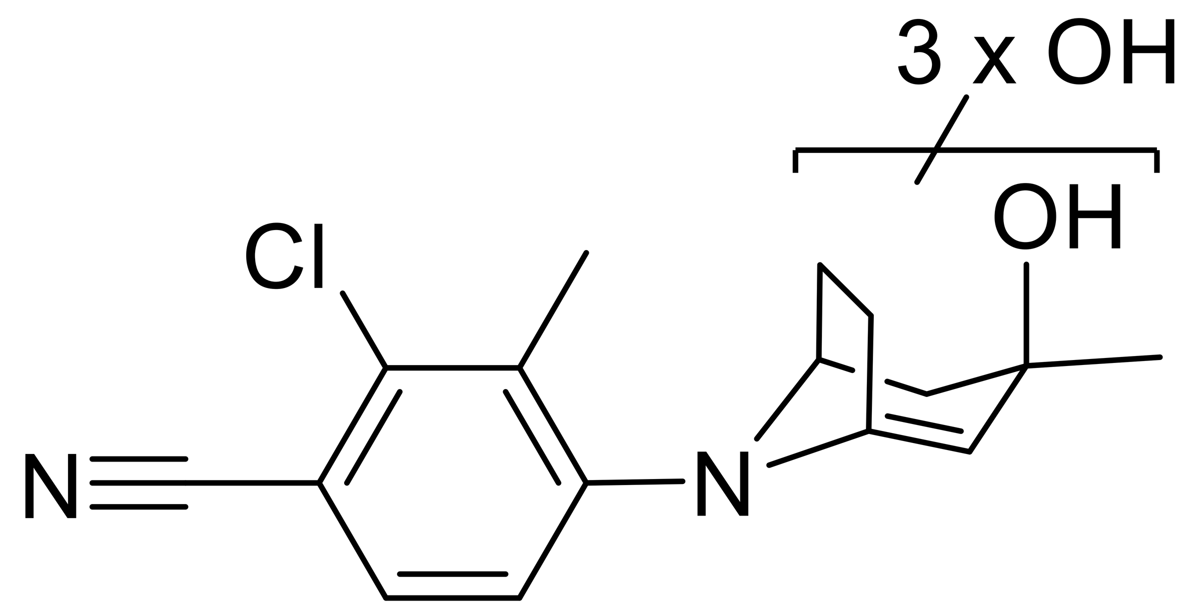

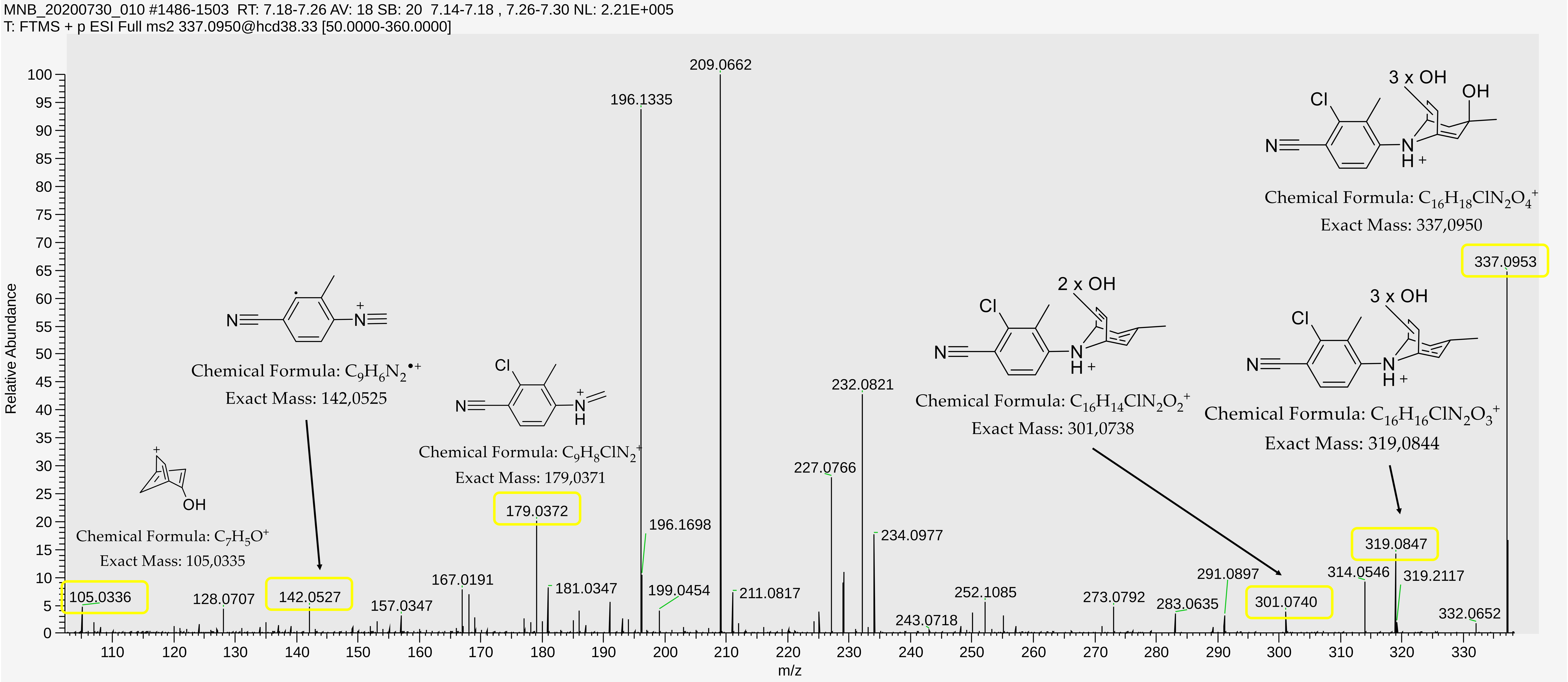

M6b

Trihydroxylation + loss of 2H
Retention time: 7.47 min
m/z: 337.0951

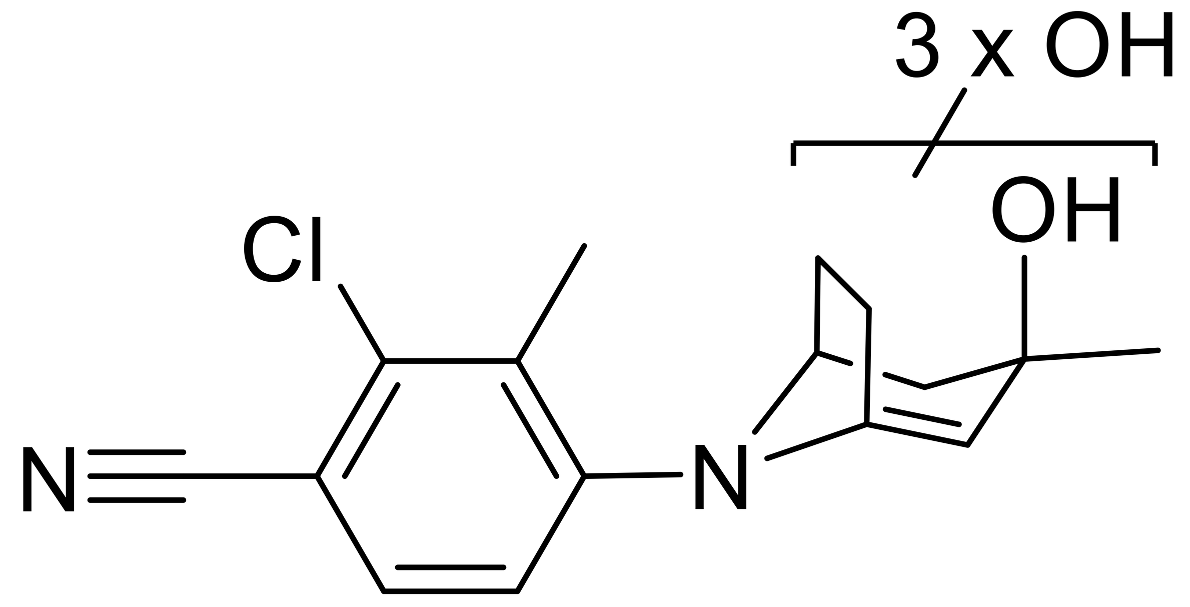

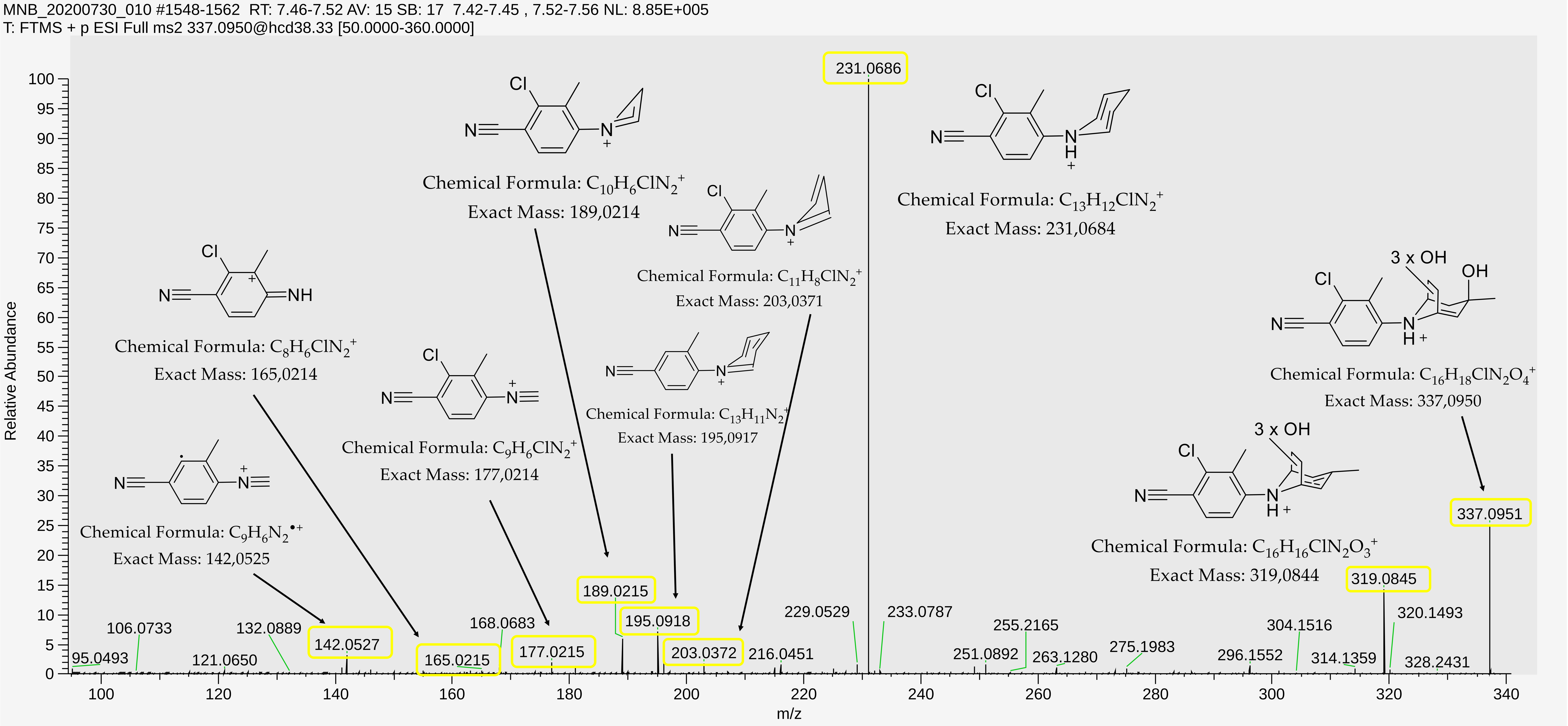

M6c

Trihydroxylation + loss of 2H
Retention time: 9.04 min
m/z: 337.0955

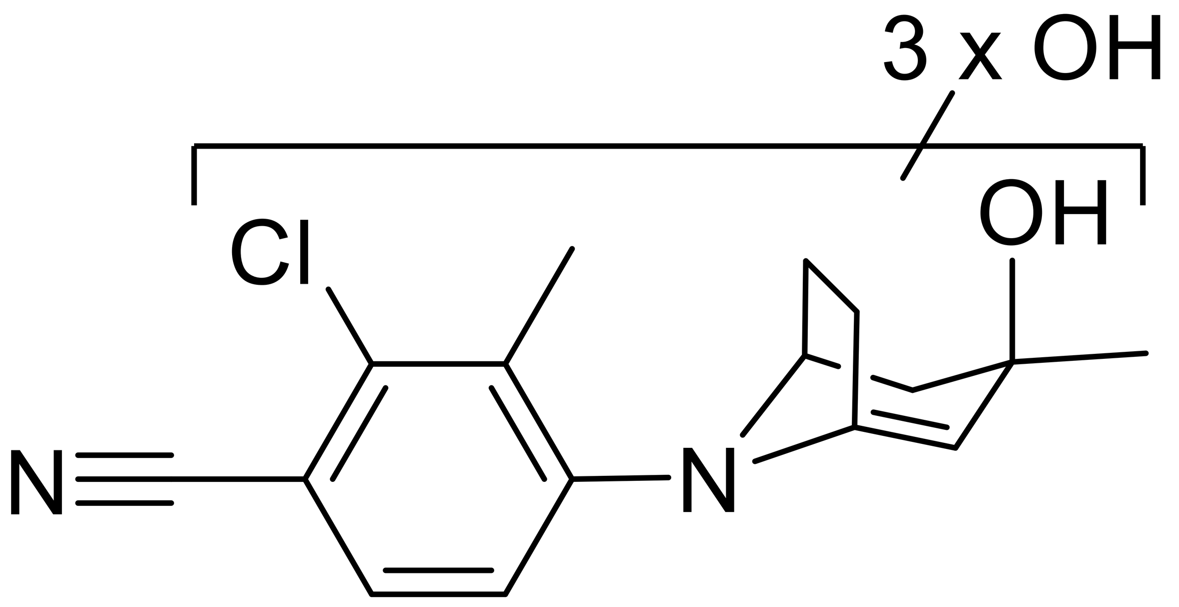

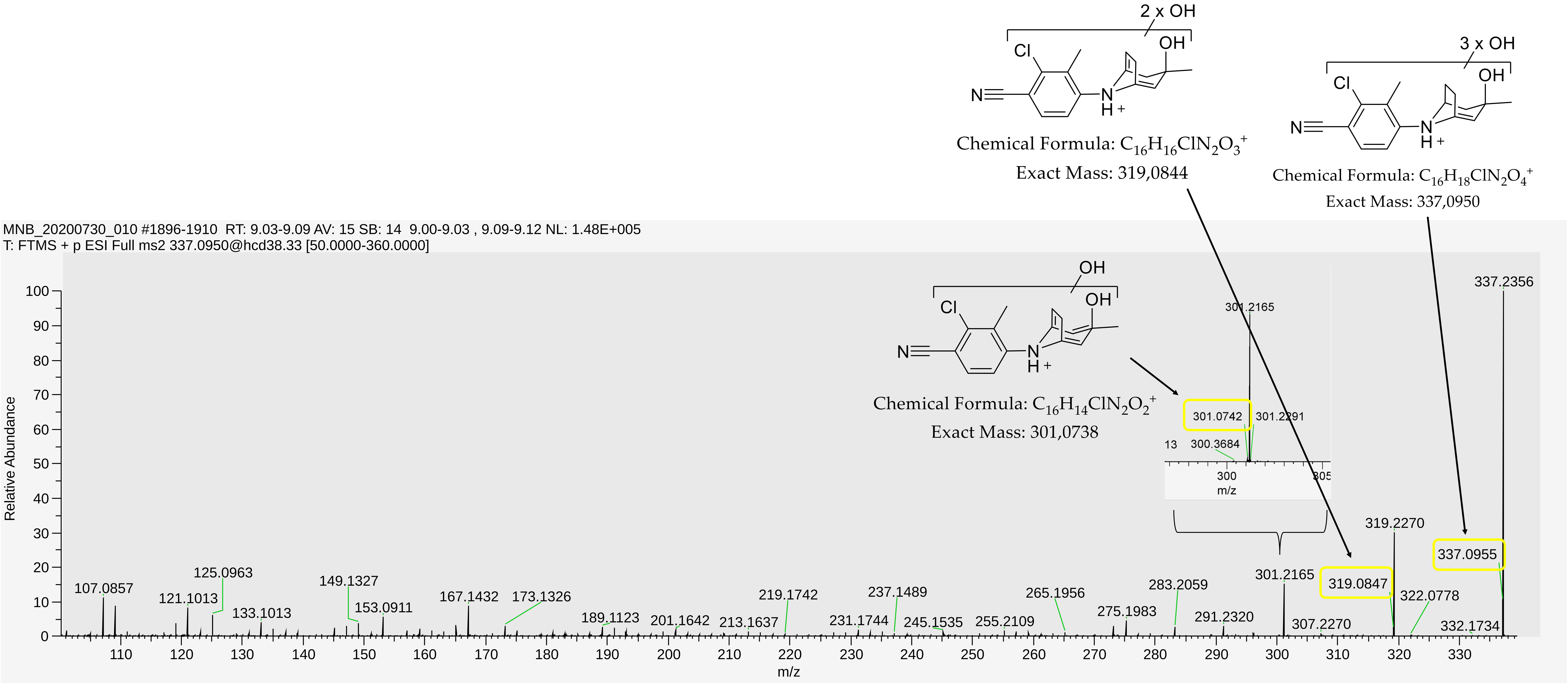

M7

Glucuronidation

Retention time: 8.95 min

m/z: 467.1580

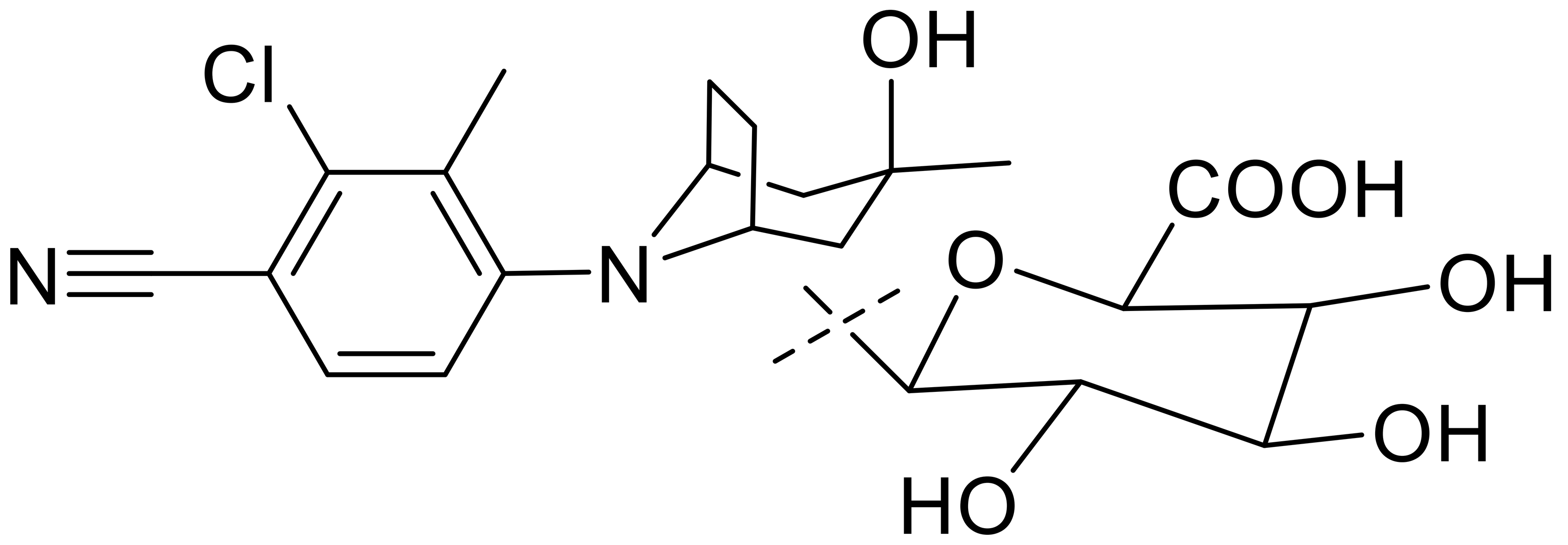

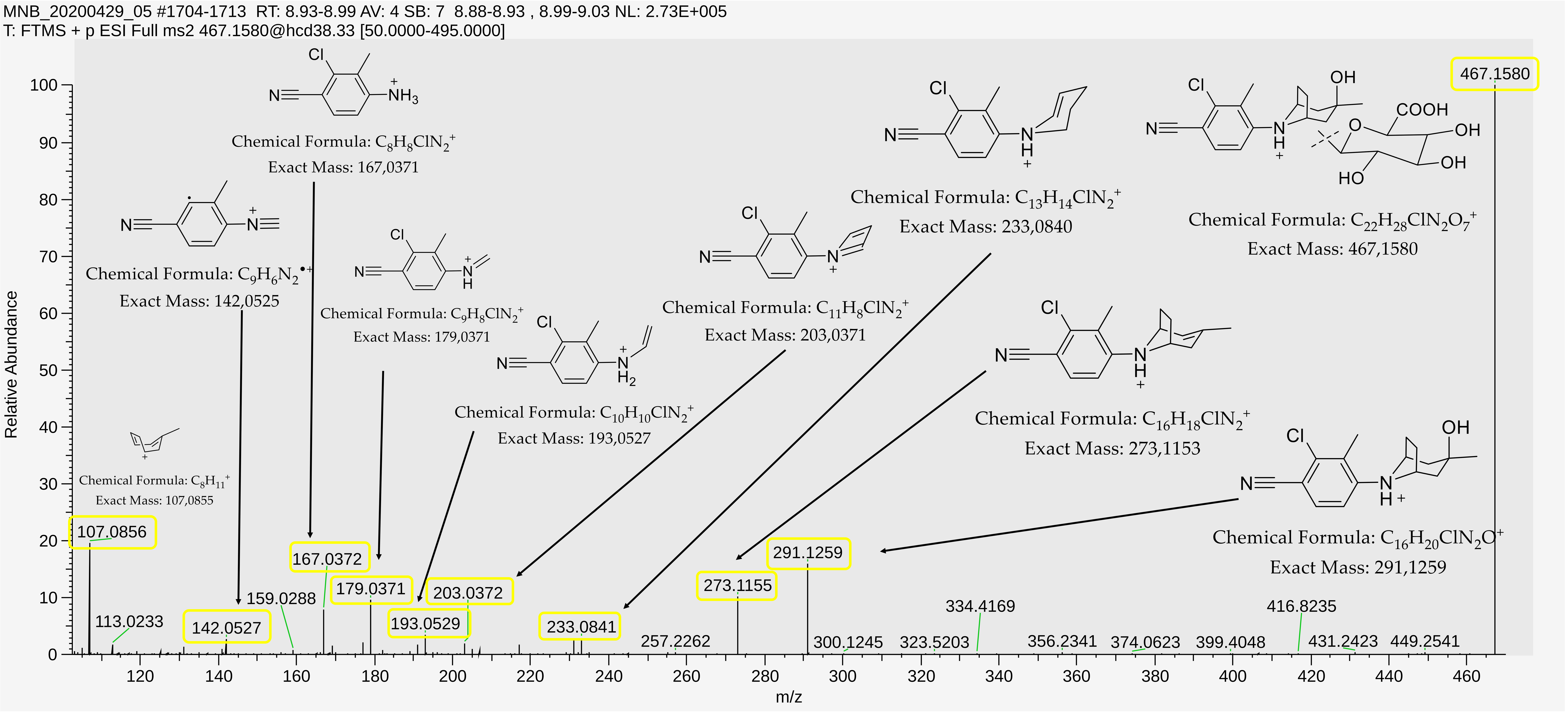

M8a

Monohydroxylation + glucuronidation
Retention time: 7.13 min
m/z: 483.1531

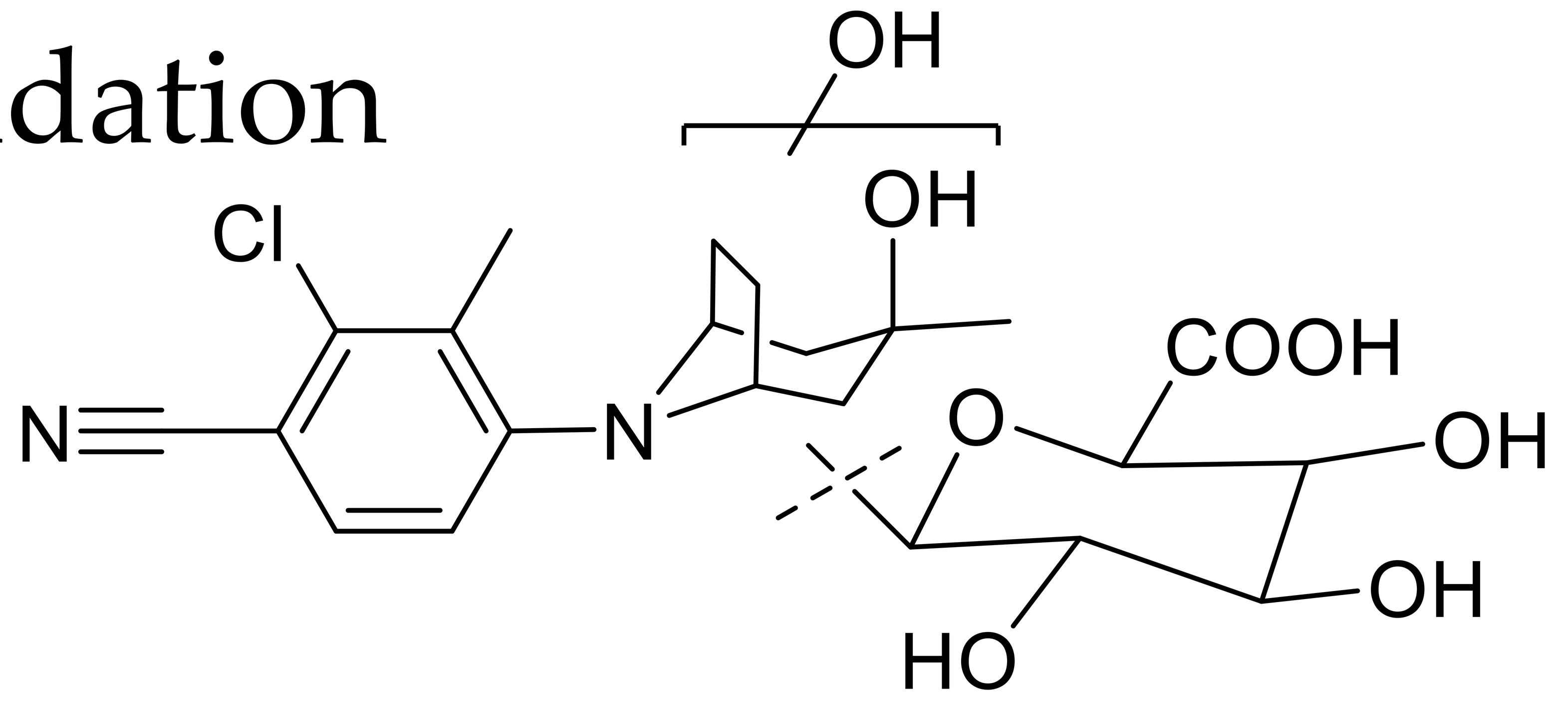

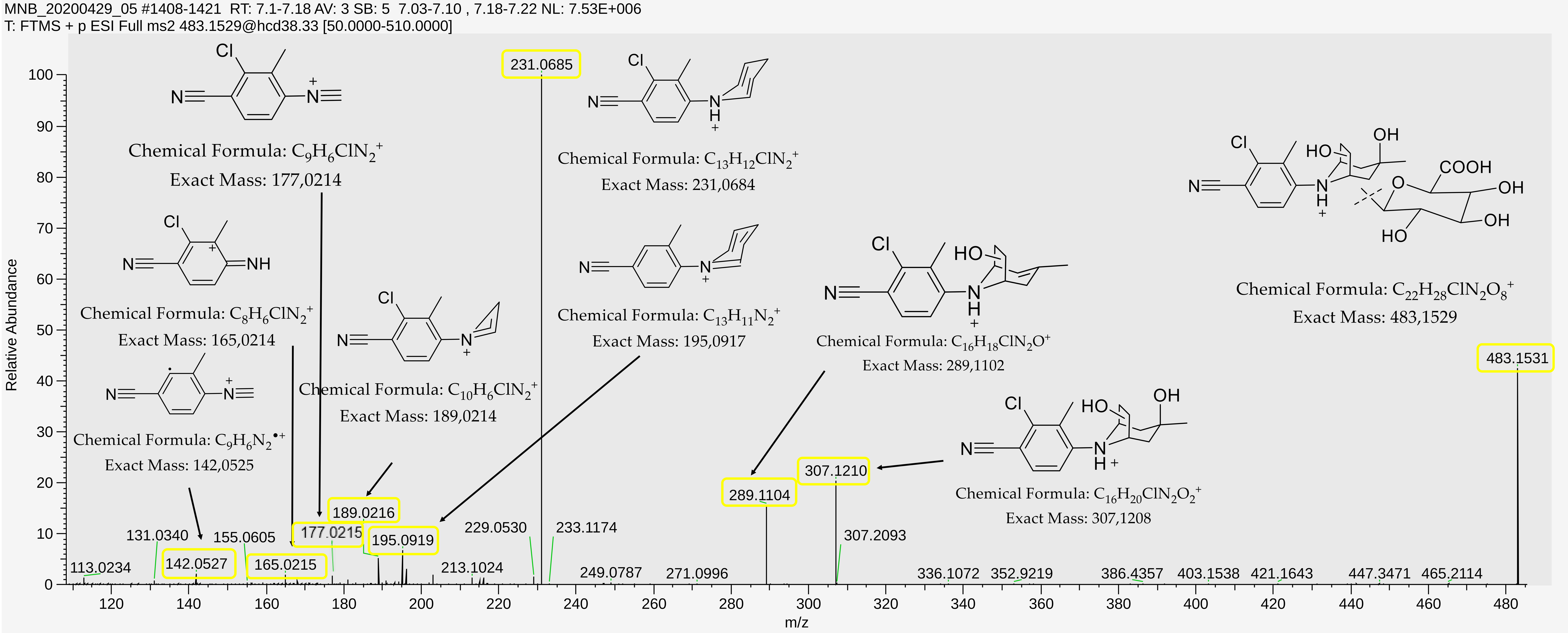

M8b

Monohydroxylation + glucuronidation
Retention time: 7.61 min
m/z: 483.1527

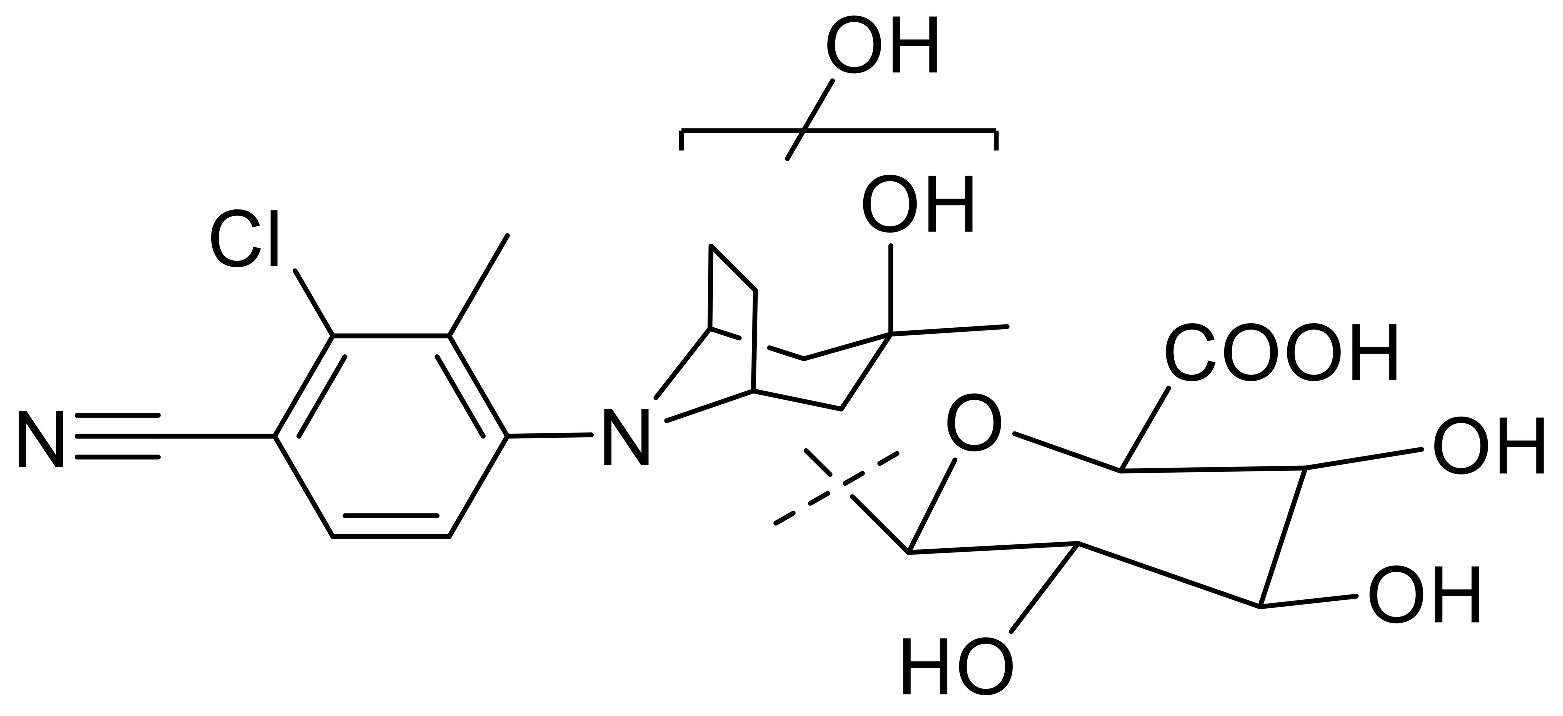

Very low intensity of remaining m/z 483.1527 and co-eluting with other ions and thereby not visible in spectrum

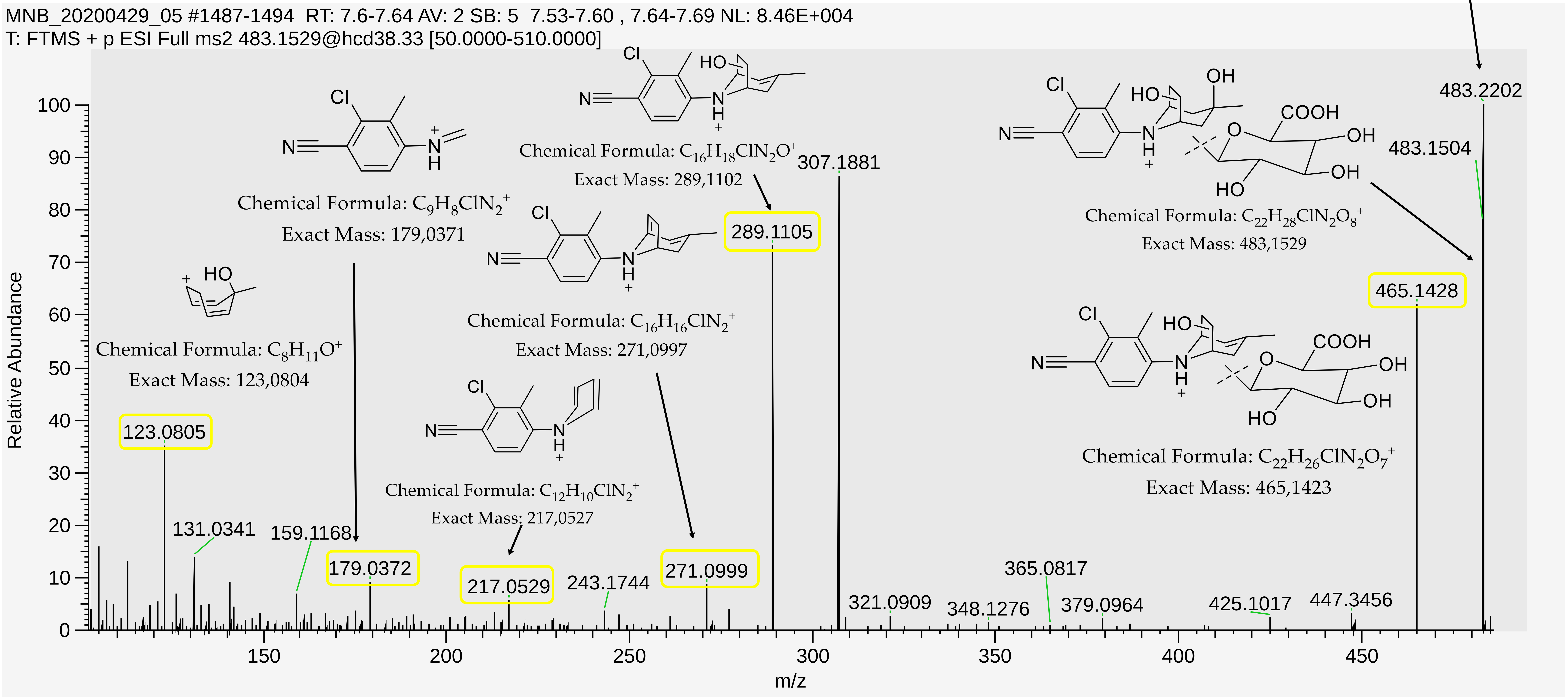

M8c

Monohydroxylation + glucuronidation
Retention time: 7.85 min
m/z: 483.1524

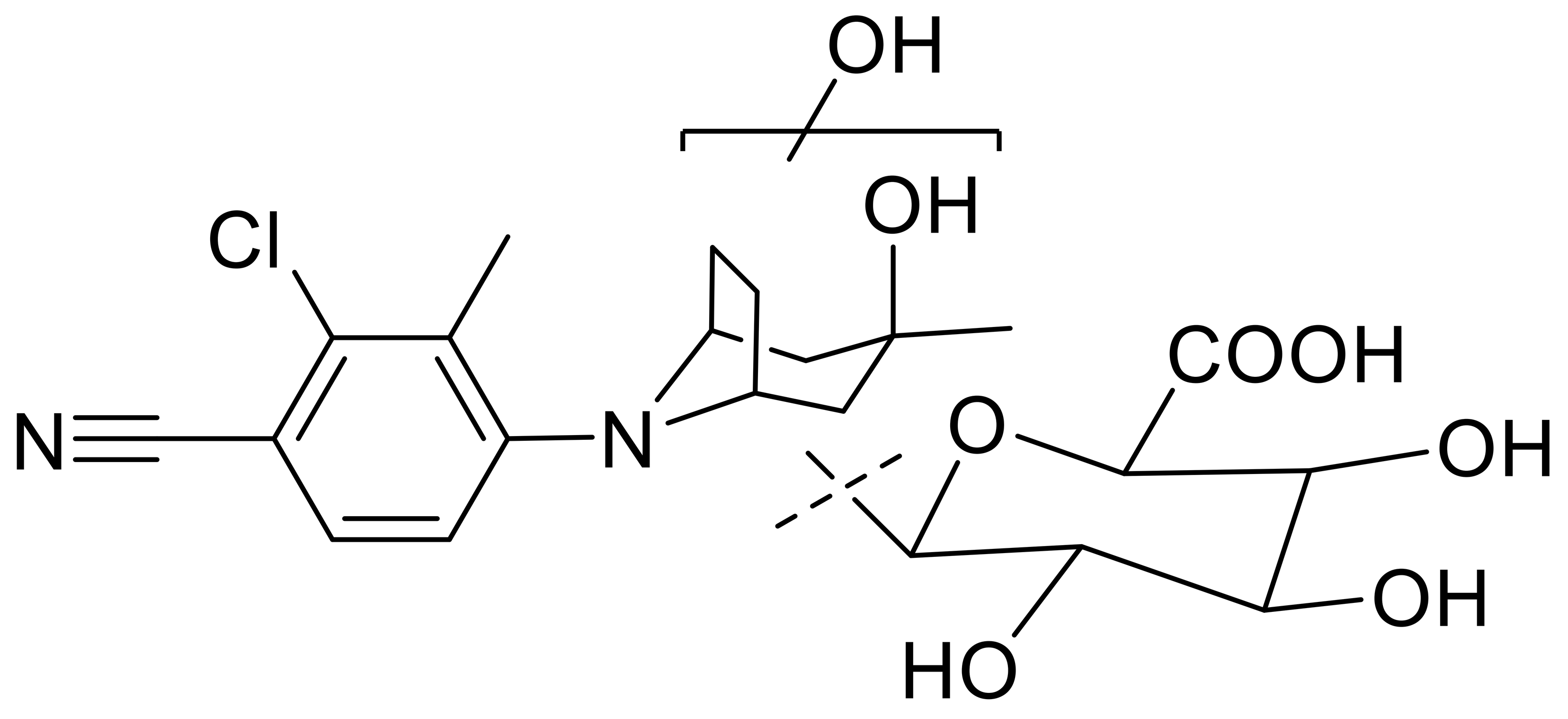

Very low intensity of remaining m/z 483.1524 and co-eluting with other ions and thereby not visible in spectrum

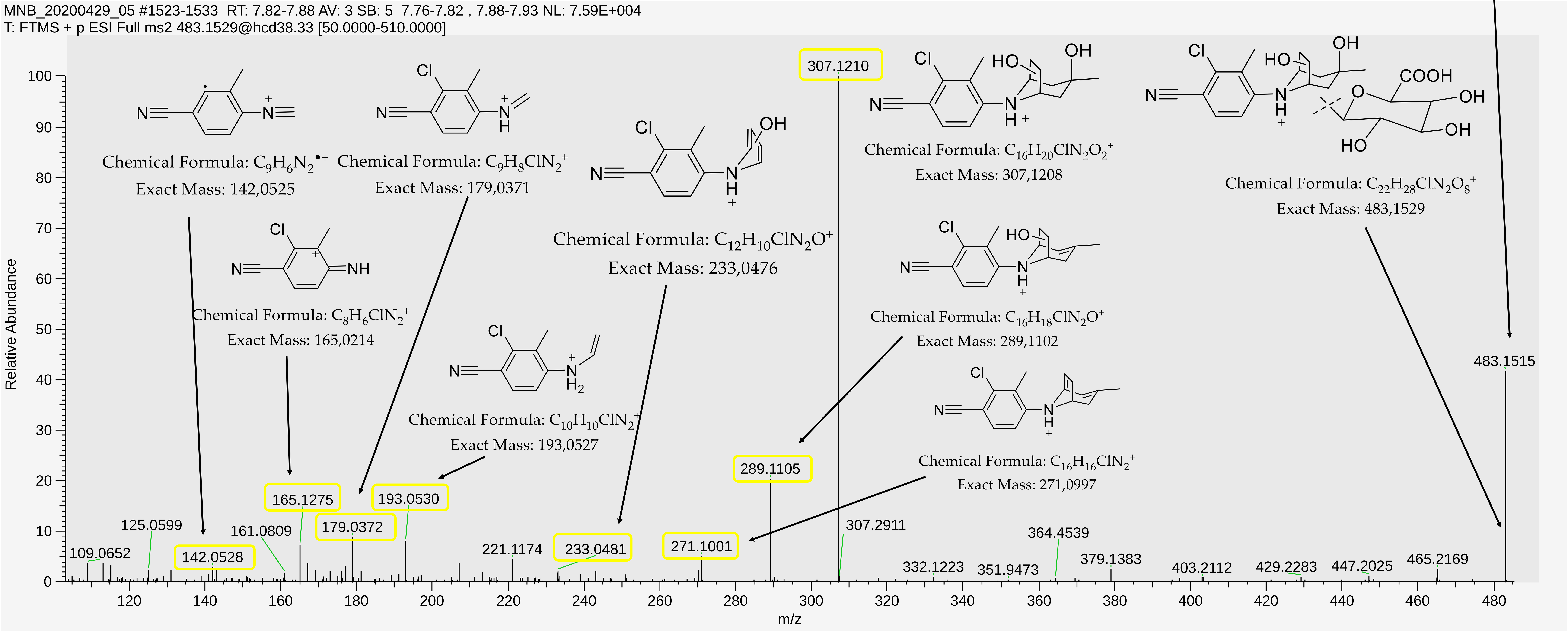

M8d

Monohydroxylation + glucuronidation
Retention time: 8.00 min
m/z: 483.1530

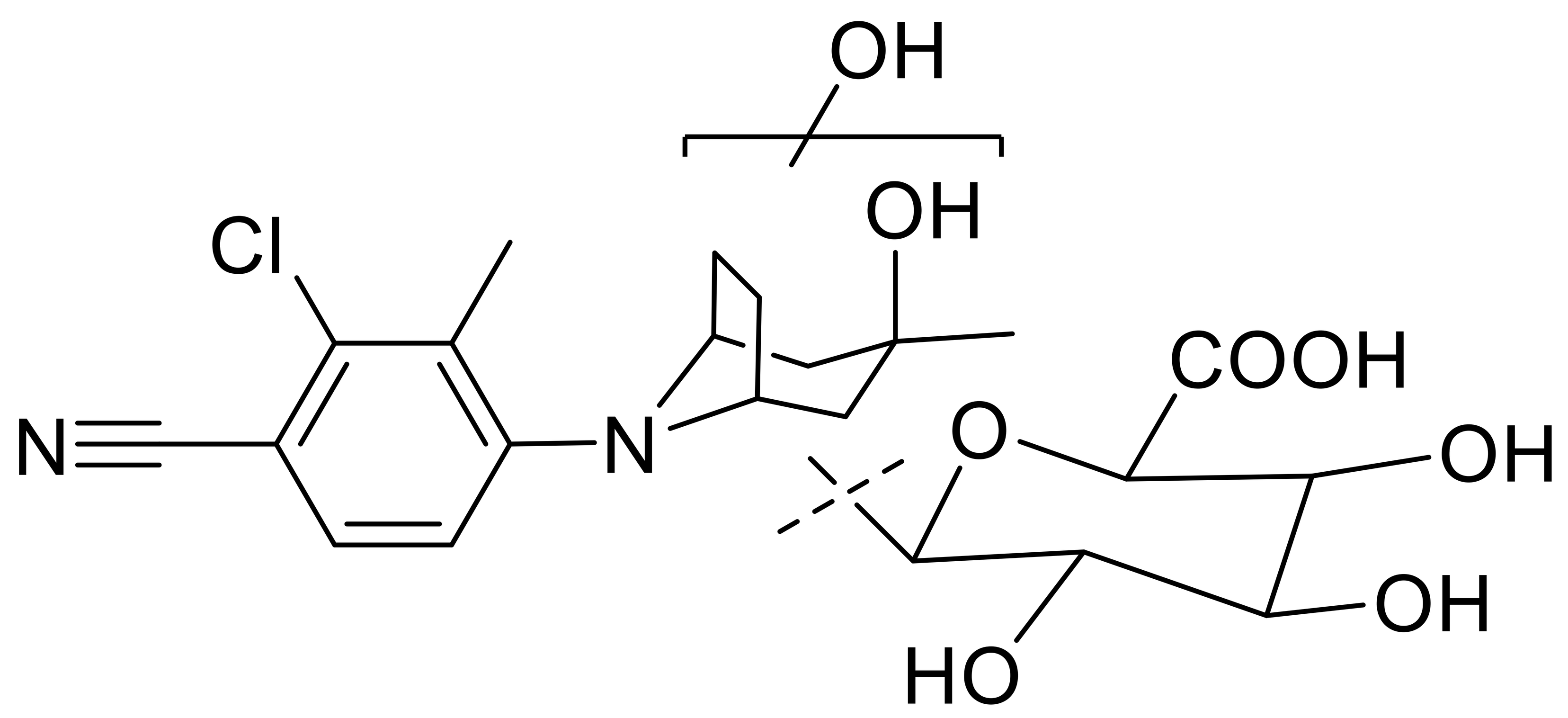

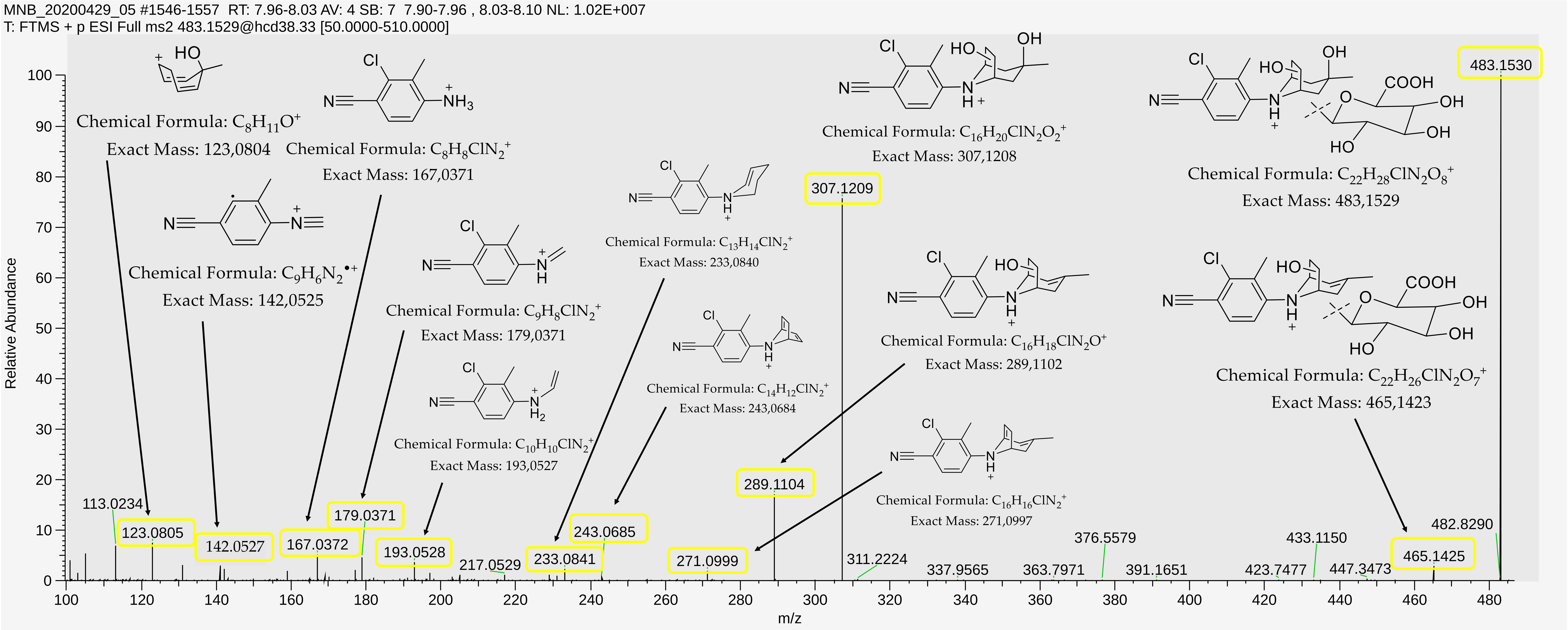

M9a

Dihydroxylation + glucuronidation
Retention time: 6.21 min
m/z: 499.1470

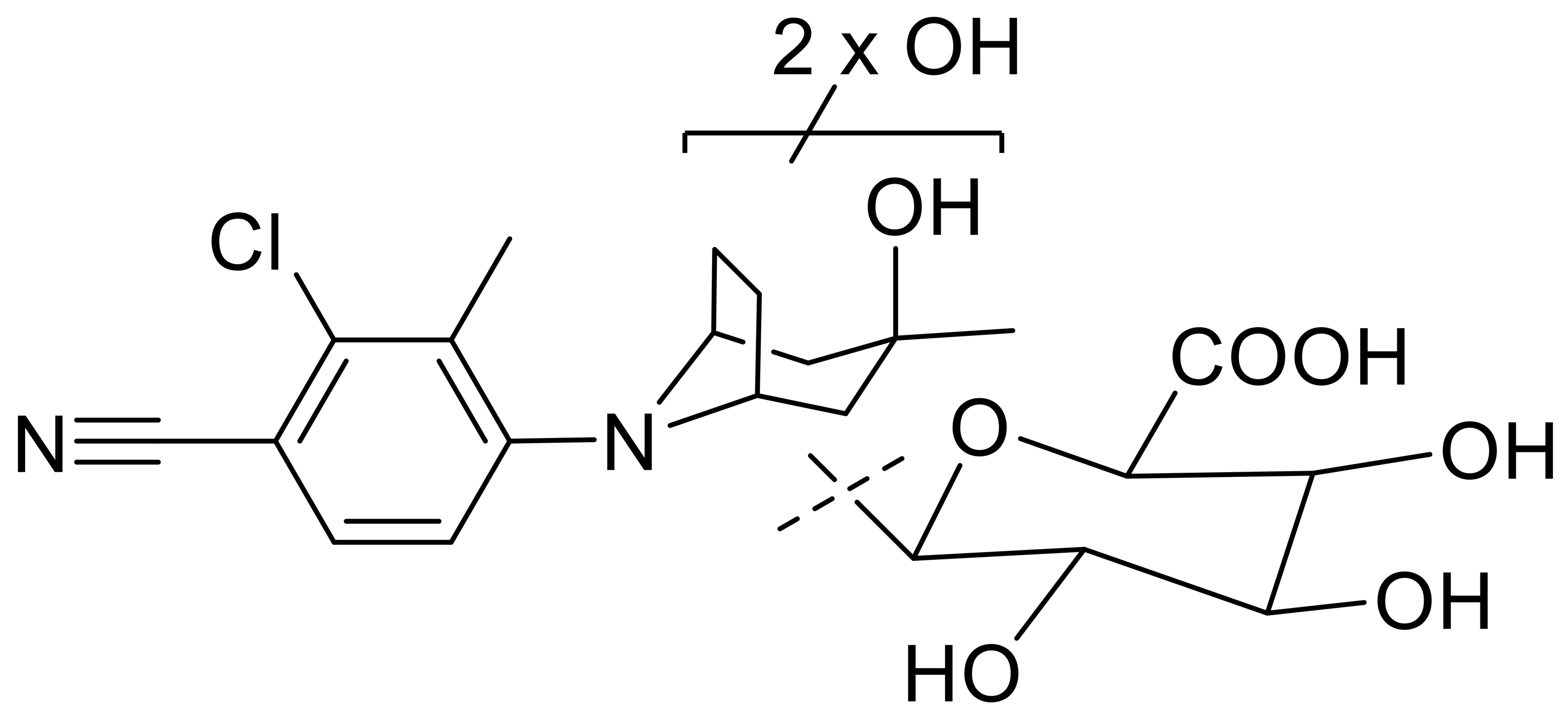

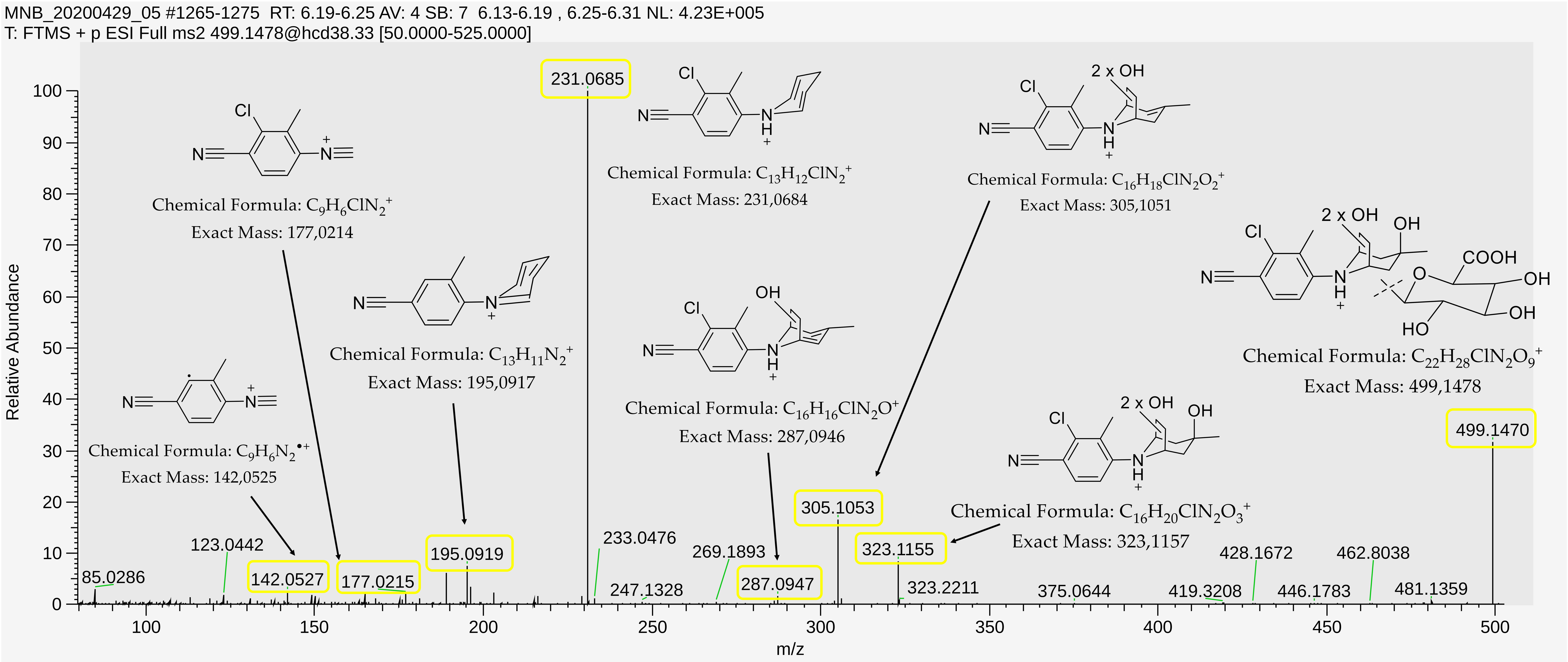

M9b

Dihydroxylation + glucuronidation
Retention time: 6.52 min
m/z: 499.1478

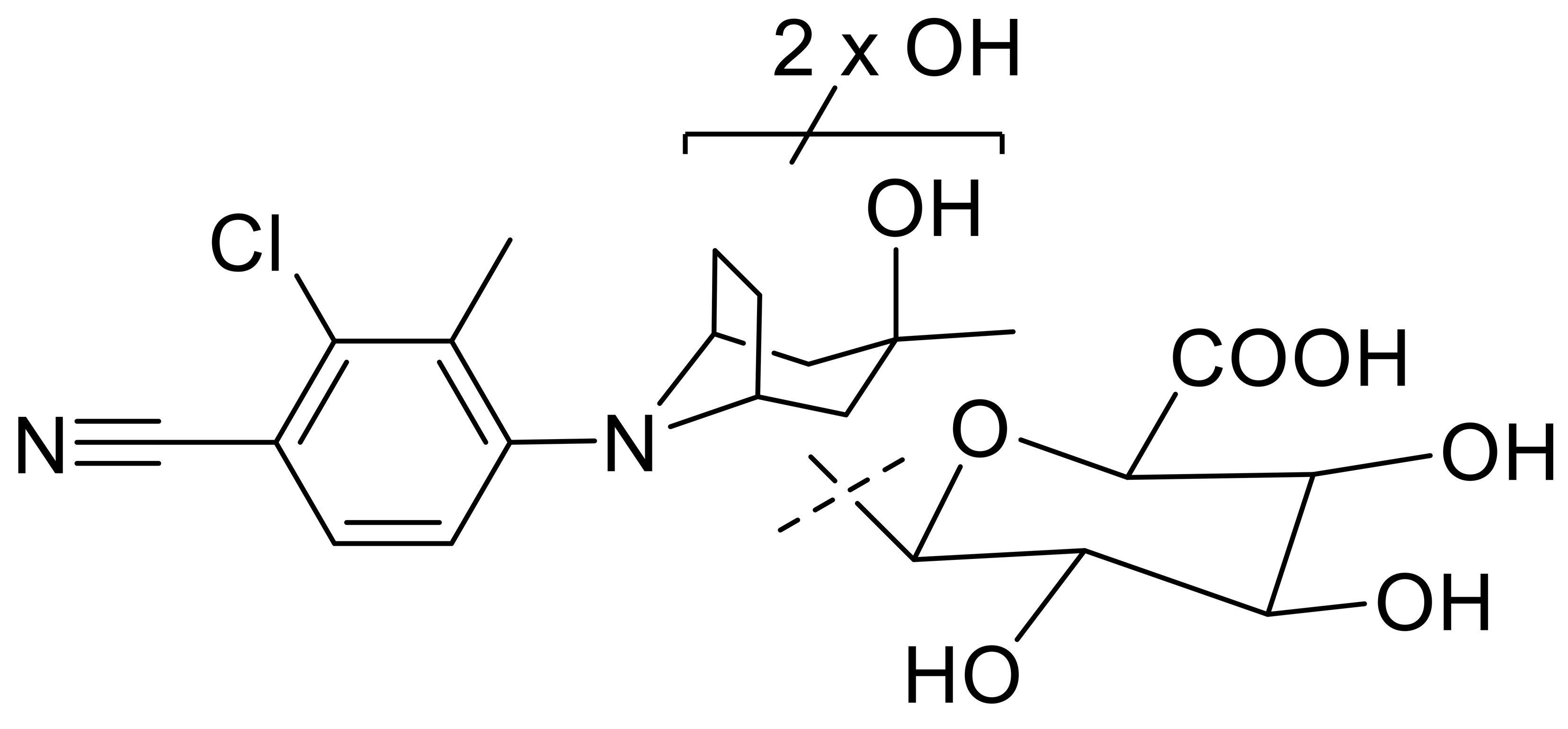

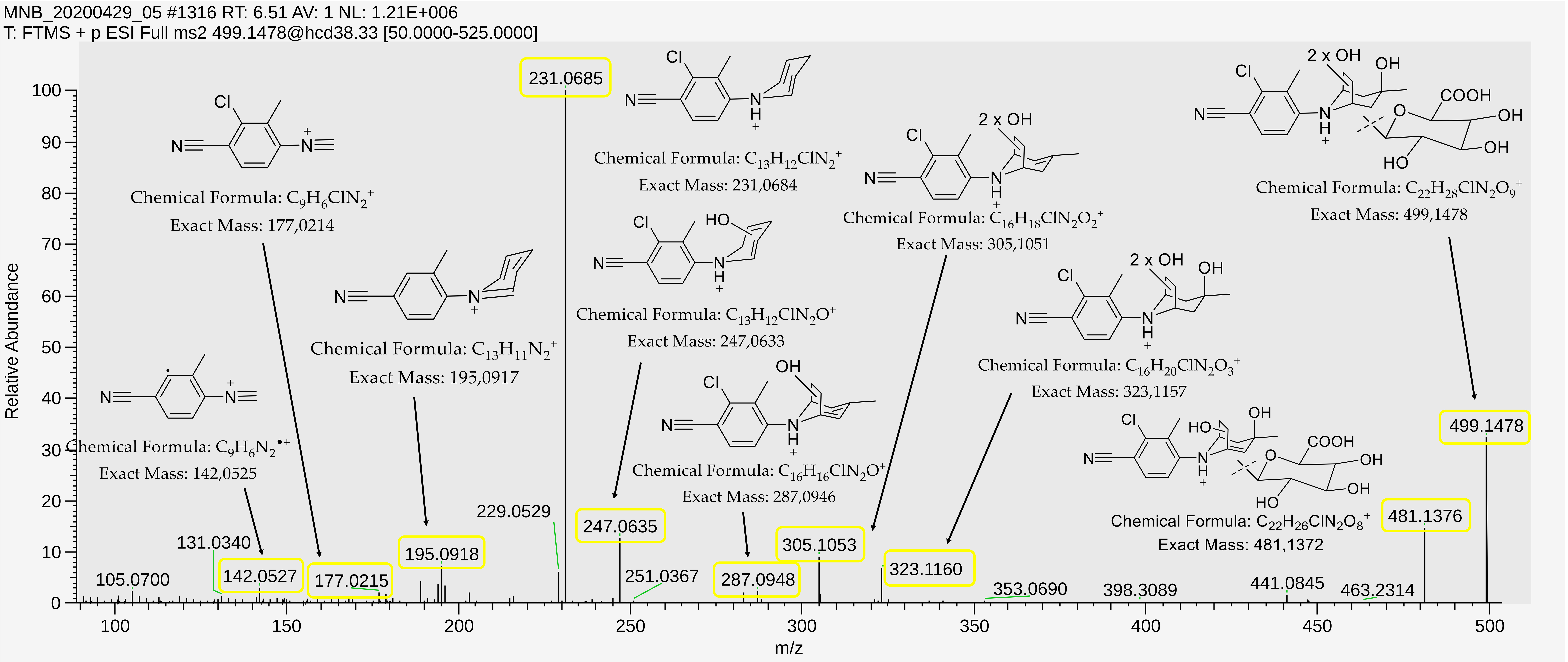

Supplement: Supplementary file 1 [file metabolites-11-00085-s001.pdf]
